# Insights into the Thermally Activated Cyclization Mechanism in a Linear Phenylalanine-Alanine Dipeptide

**DOI:** 10.1021/acs.jpcb.1c10736

**Published:** 2022-04-19

**Authors:** Laura Carlini, Jacopo Chiarinelli, Giuseppe Mattioli, Mattea Carmen Castrovilli, Veronica Valentini, Adriana De Stefanis, Elvira Maria Bauer, Paola Bolognesi, Lorenzo Avaldi

**Affiliations:** CNR-Istituto di Struttura della Materia (CNR-ISM), Area della Ricerca di Roma 1, Monterotondo Scalo 00015, Italy

## Abstract

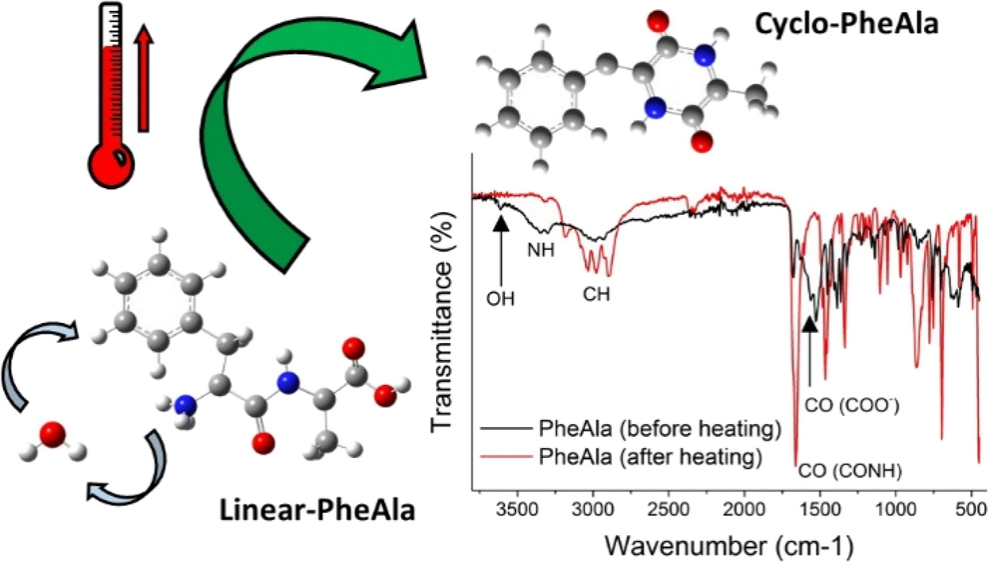

Dipeptides, the prototype peptides, exist in both linear (*l*-) and cyclo (*c*-) structures. Since the first mass spectrometry experiments, it has been observed that some *l*-structures may turn into the cyclo ones, likely via a temperature-induced process. In this work, combining several different experimental techniques (mass spectrometry, infrared and Raman spectroscopy, and thermogravimetric analysis) with tight-binding and ab initio simulations, we provide evidence that, in the case of l-phenylalanyl-l-alanine, an irreversible cyclization mechanism, catalyzed by water and driven by temperature, occurs in the condensed phase. This process can be considered as a very efficient strategy to improve dipeptide stability by turning the comparatively fragile linear structure into the robust and more stable cyclic one. This mechanism may have played a role in prebiotic chemistry and can be further exploited in the preparation of nanomaterials and drugs.

## Introduction

Peptides, *i.e.*, series of amino acids linked via peptide bonds, are one of the most important classes of biomolecules active in many relevant biological processes. Their role in proteins and enzymes and their use in the development of innovative preparation methods of nanomaterials^1−3^ have made these compounds the object of widespread interest since the 50s of the previous century.

**Figure 1:**
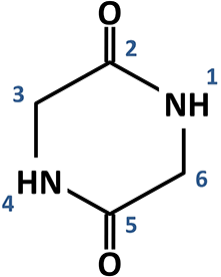
Schematic of the DKP ring.

A linear dipeptide, the simplest peptide, is made of two amino acids joined via a CO–NH peptide bond between their respective −COOH and −NH_2_ terminal groups, with the elimination of a water molecule. Cyclo dipeptides consist of a six-membered ring containing two head–tail peptide bonds. The simplest member is cyclo(glycylglycine) or diketopiperazine, DKP (Figure 1).

Thanks to properties like good binding affinity to DNA and proteins, target selectivity, and low toxicity, cyclo peptides have been considered for the development of therapeutics,^4^ too. It has also been proposed that cyclo dipeptides may have played a role in the emergence of life in the early universe,^5^ thanks to both their capability to withstand radiation and to produce crucial intermediates for the development of peptide chains.^6^ Therefore, studies of cyclo dipeptides, their formation and stability, are particularly relevant and cross-cutting.

Different methods have been exploited for the synthesis of cyclo dipeptides from solution^7^ as well as solid phase.^8^ These procedures may require several reaction steps involving precursors, catalysts, and solid supports. It has been found that the formation of cyclo dipeptides by temperature-induced cyclization of linear dipeptides in water solution results in a high yield of cyclo species,^9−11^ as water can act as a mediator favoring the head-to-tail cyclization of the linear dipeptide. On the other hand, the solid-phase synthesis can offer some advantages, such as the possibility to isolate single molecules on a solid substrate avoiding tedious purification steps^8^ and the possibility to grow peculiar nanostructures.^12^ However, this methodology usually needs *ad hoc* polymeric supports and suffers from slower reaction rates.

Since the first reports,^13,14^ it has been generally recognized through mass spectrometry techniques that the sublimation of linear dipeptides may lead to the cyclization of the compound. This thermally induced cyclization has been confirmed by further reports,^15−17^ and it is mentioned also in more recent studies.^18,19^ However, a clear understanding of the process, combining measurements and computational studies, is still lacking, mainly due to the competition between thermal decomposition and cyclization reactions,^20−23^ with the former often dominating.

Considering that thermal cyclization may have provided an effective survival mechanism for oligopeptides in harsh conditions of the primordial universe and can represent a useful resource in the case of thermal processes used for nanomaterial production,^2,12,24,25^ in this work, we investigate the structural changes of the linear l-phenylalanyl-l-alanine (*l*-PheAla) dipeptide exposed to controlled temperature in ultrahigh-vacuum (UHV) conditions and consider few other dipeptide samples to support the data interpretation.

Our study combines measurements with different techniques and theoretical simulations. In detail, we performed time-of-flight mass spectrometry (TOF-MS) of gas-phase, sublimated, samples; thermogravimetric analysis (TGA) of the pristine samples; and infrared (IR) and Raman spectroscopies of the pristine samples and of the residual ones after the sublimation for the TOF-MS experiments. The experimental results are supported and complemented by a multilevel computational protocol rooted on tight-binding and ab initio simulations, including the calculation of IR and Raman spectra, of the reaction paths and barriers of dipeptide cyclization. This study shows that a clear evolution from *l*- to *c*-structures, driven by temperature, occurs in the condensed phase. This temperature-induced cyclization in *l*-PheAla is also confirmed by a comparative spectroscopic study with cyclo-glycylphenylalanine (*c*-GlyPhe) and cyclo-alanylglycine (*c*-AlaGly).

## Experimental and Theoretical Methods

### Samples

Linear l-phenylalanyl-l-alanine (*l*-PheAla, C_12_H_16_N_2_O_3_, CAS 3918-87-4, *m* = 236 amu), cyclo (3*S*)-3-benzyl-2,5-piperazinedione (*c*-GlyPhe, C_11_H_12_N_2_O_2_, CAS 10125-07-2, *m* = 204 amu), linear glycyl-l-alanine (*l*-GlyAla, C_5_H_10_N_2_O_3_, CAS 3695-73-6, *m* = 146 amu), and linear glycyl-l-phenylalanine (*l*-GlyPhe, C_11_H_14_N_2_O_3_, CAS 3321-03-7, *m* = 222 amu) molecules were purchased from Sigma-Aldrich, while cyclo (3*S*)-3-methyl-2,5-piperazinedione (*c*-AlaGly, C_5_H_8_N_2_O_2_, CAS 4526-77-6, *m* = 128 amu) was purchased from BACHEM. All species had a purity ≥95% and were used without further purification.

### TOF-MS Measurements

The mass spectra were collected using a custom-made Wiley–McLaren time-of-flight mass spectrometer^26^ coupled to a VUV rare-gas discharge lamp and an effusive beam of the molecule under investigation. A general description of the apparatus was reported elsewhere.^27,28^ Briefly, the instrument was equipped with a channel electron multiplier (CEM) and microchannel plates (MCP) for electron and ion detection, respectively. The detection of a kinetic-energy unselected photoelectron provided the trigger for the measurement of the time of flight of the ions by a TDC card (Model ATMD-GPX, ACAM Messelectronic). At variance with ref (27), the effusive beam of the sample was produced by a stainless-steel oven that housed a crucible to sublimate solid samples and was heated by a twin-core Thermocoax. The mass spectra were collected at a 21.22 eV (He I) photon energy, in repeated acquisitions of 10′ per spectrum to monitor the temporal evolution of the sublimation. The samples, which were powders at standard ambient temperature and pressure, were sublimated at 110–130 °C, depending on the sample. The base pressure of the TOF-MS was 1–2 × 10^–8^ mbar, which increased to 4–5 × 10^–7^ mbar when the He gas discharge lamp was in operation.

### Thermogravimetric and Differential Thermal Analysis (TG-DTA)

The TG-DTA measurements were performed on approximately 13 mg of the sample set in an alumina crucible using a Stanton Redcroft STA 1500 instrument. The sample was heated up to 800 °C in a pure nitrogen atmosphere (99.9995%), with heating and flow rates of 5 °C/min and 50 mL/min, respectively.

### IR and Raman Spectroscopies

The IR measurements were performed using an FT-IR Shimadzu Prestige 21 equipped with an ATR (attenuated total reflection) Golden Gate accessory (Specac), a Michelson interferometer, and a DLATGS detector. The acquisition range and the resolution were 4000–450 and 4 cm^–1^, respectively.

A Dilor XY spectrometer, with a 514.5 nm excitation wavelength and a 2.5 mW power, was used to perform Raman spectroscopy. The Raman spectra were obtained from 5 scans of 240 s in the spectral range from 180 to 3220 cm^–1^.

The IR and Raman spectra were acquired at room temperature on both the pristine sample and the residual sample after heating in an oven at the working temperature during mass spectrometry measurements. In the following, these two samples will be named *l*-PheAla and *r*-PheAla for short.

The TOF-MS, IR, and Raman measurements were carried out on three different *l*-PheAla samples to secure the reproducibility of the results.

### Theoretical Methods

Theoretical simulations of harmonic IR and Raman spectra were performed by following a multilevel protocol, applied to isolated molecules embedded in a dielectric environment. A preliminary screening of molecular configurations was performed using a conformer-rotamer ensemble sampling tool (CREST), which uses the GFN2-xTB Hamiltonian as an “engine” for the exploration of potential energy surfaces in order to find low-energy structures.^29−31^ In the case of neutral linear (*l*-neutral) and cyclo (*c*-neutral) dipeptides, the most stable structures were used as the initial guess for DFT geometry optimizations. In the case of the zwitterionic linear structure (*l*-zwitterion), the extremely strong interaction between the terminal charged −NH_3_^+^ and COO^–^ groups led to the occurrence of spurious vibrational normal modes. In this case, the starting configuration of the molecule for DFT calculations was cut out from the hydrated *l*-PheAla crystal structure.^32^ DFT calculations were performed using the ORCA suite of programs.^33,34^ Molecular structures were fully reoptimized using the B3LYP functional^35^ and the D3BJ dispersion correction.^36^ Kohn–Sham orbitals were expanded on the def2-QZVPP Gaussian basis set.^37^ The corresponding def2/J basis was also used as an auxiliary basis set for Coulomb fitting in a resolution-of-identity/chain-of-spheres (RIJCOSX) framework. Cyclization mechanisms were investigated by using the same multilevel protocol. Reaction paths were first systematically explored by using an automated reaction-path finder based on tight-binding simulations.^30^ Representative paths were used as the initial guess for climbing image nudged elastic band (CI-NEB) calculations based on density functional theory. As suggested in previous calculations of symmetric aliphatic dipeptides,^38^ accurate estimates of reaction barriers along different cyclization paths were obtained using the M06-2X functional^39^ and a def2-TZVPP basis set.

## Results

**Figure 2:**
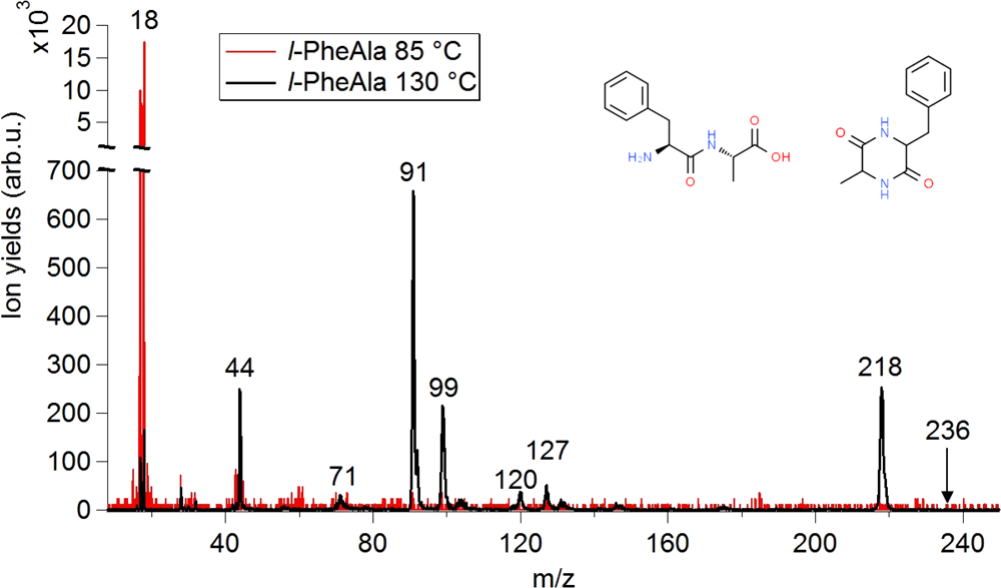
Photoionization mass spectra of *l*-PheAla measured at 85 (red line) and 130 °C (black line) at 21.22 eV incident radiation. The spectra have been normalized to the same acquisition time. In the inset, the schematic structures of *l*-PheAla and *c*-PheAla are shown. The *m*/*z* value of the main fragments is indicated, while the proposed assignments are reported in Table 1.

**Figure 3:**
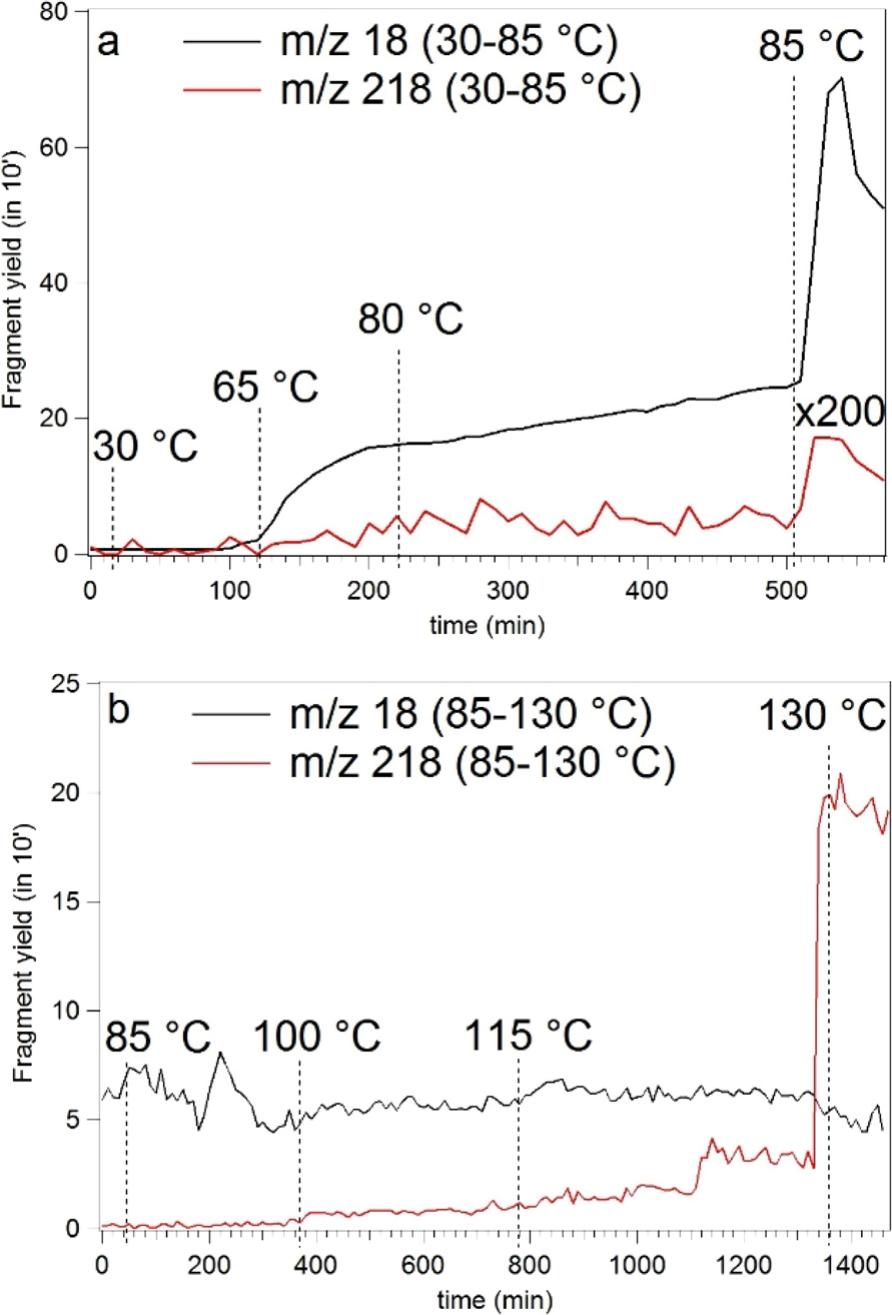
(a,b) Yields of *m*/*z* 18 (black line) and 218 (red line) fragment ions as a function of temperature and acquisition time. The measurements in panel (b) have been performed after leaving the sample at 85 °C for about 12 h.

The TOF mass spectra of *l*-PheAla were acquired by slowly increasing the temperature in steps of 5 °C every 100 min. In Figure 2, the mass spectra measured at 85 and 130 °C are shown. In the spectrum collected at 85 °C, only ions at *m*/*z* 18 and 17, related to water and its OH^+^ fragment, are observed, while at 130 °C, a series of dipeptide fragments (see Table 1) are detected. At this temperature, the highest *m*/*z* ratio is 218 with no trace of the *l*-PheAla parent ion (*m*/*z* 236) observed. In Figure 3, the yields of *m*/*z* 18 and 218 ions are reported versus time, while the temperature of the crucible is increased. The water ion yield displays a nonlinear trend with a drastic increase at 85 °C (Figure 3a), which slowly decreases over several hours with the temperature fixed at 85 °C in UHV conditions (data not shown); after this stage, a temperature increase up to 130 °C does not result in any further increase (Figure 3b).

**Table 1:** Proposed Assignment of the Main Features in the Mass Spectrum of *l*-PheAla Acquired at 130 °C and Shown in Figure 2

*m*/*z*	assignments
17 and (18)	OH^+^ and (H_2_O)^+^
44	C_2_H_6_N^+^
71	C_4_H_9_N^+^ and C_3_H_5_NO^+^
91	C_7_H_7_^+^
99	C_4_H_7_N_2_O^+^
120	C_8_H_10_N^+^
127	C_5_H_7_N_2_O_2_^+^
218	[(*l*-PheAla)–H_2_O]^+^

Despite its intensity being orders of magnitude smaller, the *m*/*z* 218 yield displays a trend similar to the one detected for *m*/*z* 18 in the temperature range of 30–85 °C (Figure 3a), with a significant increase also of lighter fragments (data not shown) at about 125 °C. Stabilizing the oven temperature at 130 °C, no further changes in the relative intensities of these fragments are observed after 24 h. Further heating up to 160 °C results in increased gas density, but this does not result in appreciable changes in the TOF mass spectrum, suggesting a good thermal stability of the sample, at least over short times. However, for this work, we selected 130 °C as the working temperature in order to limit contaminations of the apparatus and to prevent possible decomposition of the sample over long times. The *l*-PheAla parent ion (*m*/*z* 236) was never observed throughout the whole heat-up process.

With support from the literature,^13,40,41^ the main features of the mass spectrum in Figure 2 have been assigned (Table 1) to the parent cation minus water (*m*/*z* 218) and amine fragments [C_7_H_7_CH–NH_2_]^+^ and [CH_3_CH–NH_2_]^+^ (*m*/*z* 120 and 44, respectively); the [C_5_H_7_N_2_O_2_]^+^ and its complementary fragment C_7_H_7_^+^ (*m*/*z* 127 and 91, respectively) may be attributed to the loss of the Phe and Ala residues, respectively, from *m*/*z* 218; the fragment at *m*/*z* 99 may be tentatively assigned to a further CH–CH_3_ loss from *m*/*z* 127.

Since the pioneering mass spectrometry studies of dipeptides,^14^ it has been realized that some cyclization may occur at the typical sublimation temperature^13^ (120–160 °C). According to these reports, evidence of the process is provided by the observation of ions at (i) *m*/*z* 18, (ii) *m*/*z* corresponding to the [parent ion – water molecule], (iii) *m*/*z* corresponding to the amine fragment (RCH=NH_2_)^+^ where R is the side chain of the amino acid, and (iv) other smaller ions produced by the decomposition of the fragment following an initial elimination of CO and HNCO, as established from the study of deuterated samples at the active sites. The features observed in the present mass spectrum are consistent with the findings of ref (14) suggesting the formation of cyclic species at some stages of the experiments.

**Figure 4:**
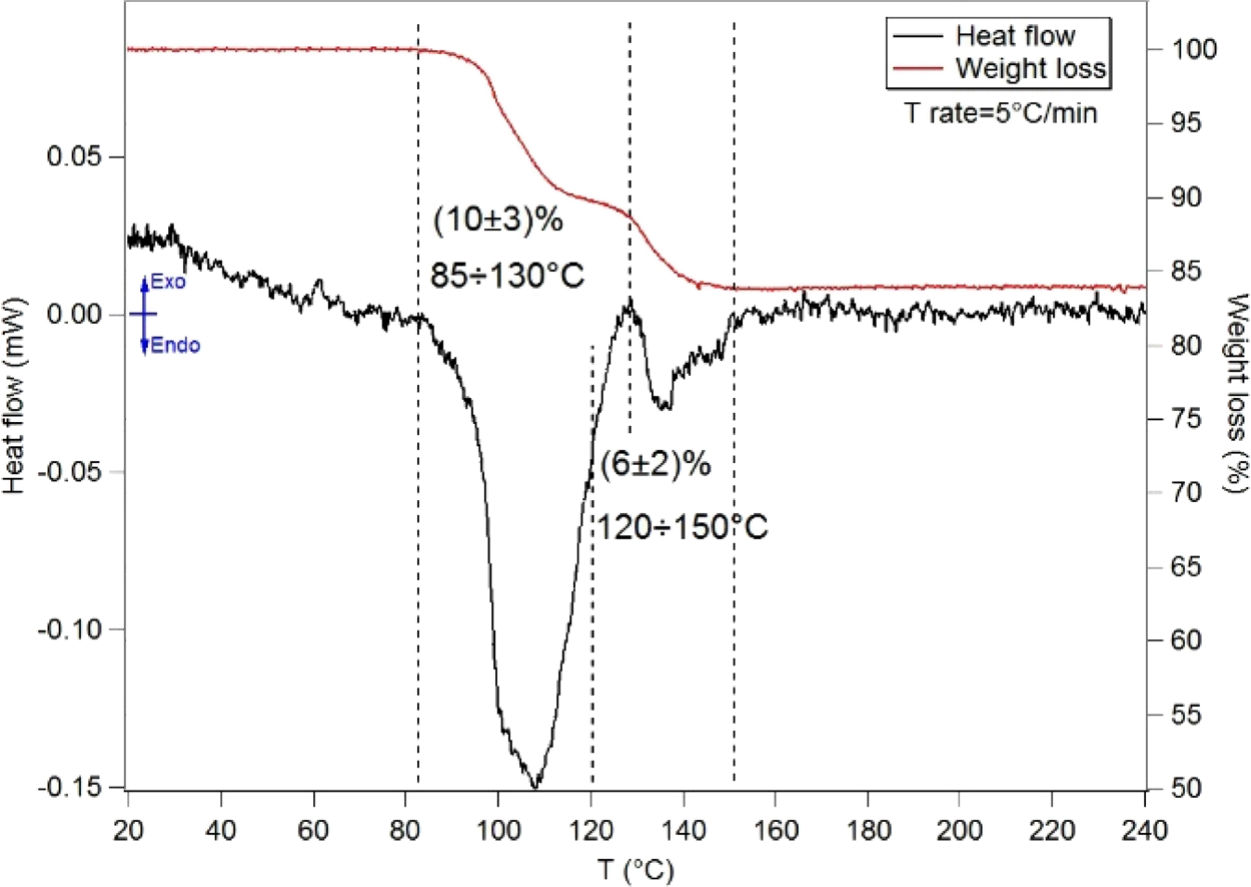
TG-DTA of *l*-PheAla (see the Experimental and Theoretical Methods for experimental details). The weight losses (%) and the corresponding temperature ranges are reported.

A thermogravimetric analysis of *l*-PheAla has been performed over a temperature range up to 800 °C. Here, we discuss the results in the range of interest for the mass spectrometry experiments (*T* < 200 °C), while the overall description is reported in the Supporting Information (SI). In the temperature range of 30–200 °C, the TG-DTA displays two endothermic peaks in the enthalpy of the system (measured as heat flow in mW) centered at about 105 and 135 °C, respectively (see Figure 4). The mass losses (%) in the temperature ranges (dashed lines) reported in Figure 4 have been estimated by a fit of the derivative of the weight curve using Voigt functions, leading to the identification of two partially overlapping desorption steps with weight losses of about 10 and 6%, respectively.

According to previous reports,^42,43^ the first mass loss of 10 ± 3% in the range of 85–130 °C can be attributed to the release of hydration water in the sample. The second step of 6 ± 2% mass loss in the range of 120–150 °C may be associated to a structural rearrangement of the sample, possibly related to the formation of amide bonds, as in the case of other linear dipeptides.^24,25,44^

With the onset temperature of evaporation/sublimation or thermal decomposition measured in a TG-DTA experiment at ambient pressure being higher than the one measured in UHV conditions,^45^ the onset temperature of the second step of mass loss (≈120 °C at ambient pressure) is expected to be lower in UHV conditions. Accordingly, the TOF-MS results in Figure 3 are consistent with the interpretation of the TG-DTA of the “outgassing” of adsorbed water (linear trend in *m*/*z* 18 intensity up to *T* ≈ 85 °C) followed by a structural reorganization involving further release of water (step at *T* ≈ 85 °C in the *m*/*z* 18 intensity).

**Figure 5:**
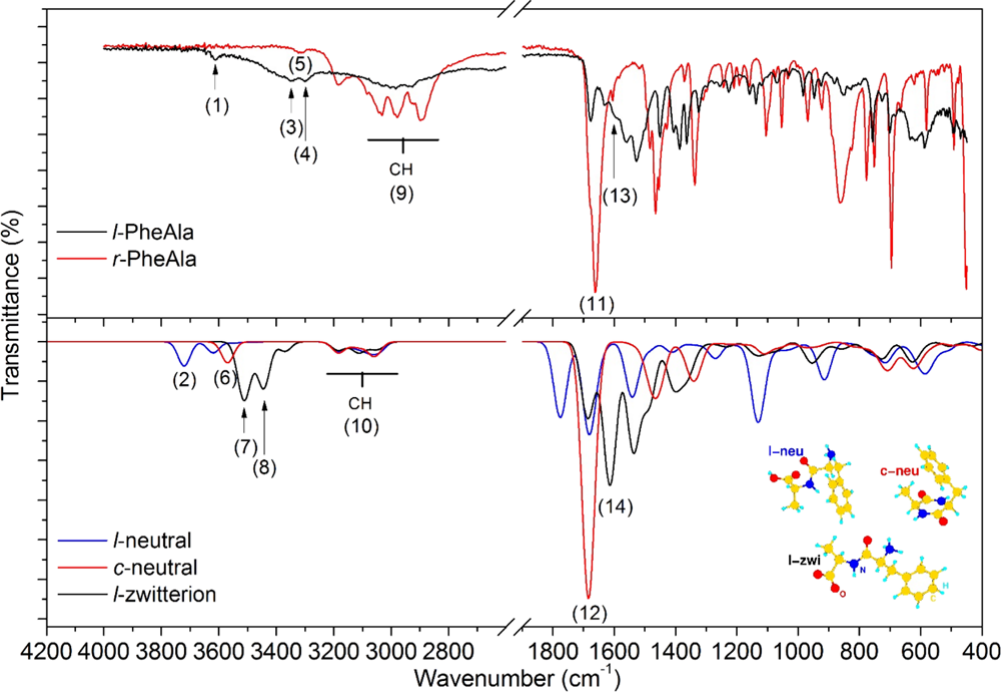
Top panel: comparison between the IR spectra of *l*-PheAla (black curve) and *r*-PheAla (red curve). Bottom panel: comparison between the simulated IR spectra of the *l*-neutral (blue), *c*-neutral (red), and *l*-zwitterion (black) PheAla. The numerical labels refer to some of the main experimental and theoretical characteristic peaks of the different species indicated in Table 2. Inset: optimized structures of *l*-neutral (*l*-neu), *c*-neutral (*c*-neu), and *l*-zwitterion (*l*-zwi) PheAla molecules.

It is well-known that IR spectroscopy can be used as a diagnostic tool of molecular structures and functional groups.^46^ Hence, to investigate the possibility of a molecular rearrangement occurring during sublimation, we recorded IR spectra of both *l*-PheAla and *r*-PheAla. These spectra, reported in the upper panel of Figure 5, clearly display significant differences.

In this regard, only *r*-PheAla shows intense and well-resolved absorptions in the functional group region of 1300–1700 cm^–1^, while for *l*-PheAla, only broad peaks centered at ≈1600 and 3300 cm^–1^can be observed. Conversely, the broad spectral feature at ≈3000 cm^–1^ in *l*-PheAla evolves in a well-resolved manifold in *r*-PheAla.

**Figure 6:**
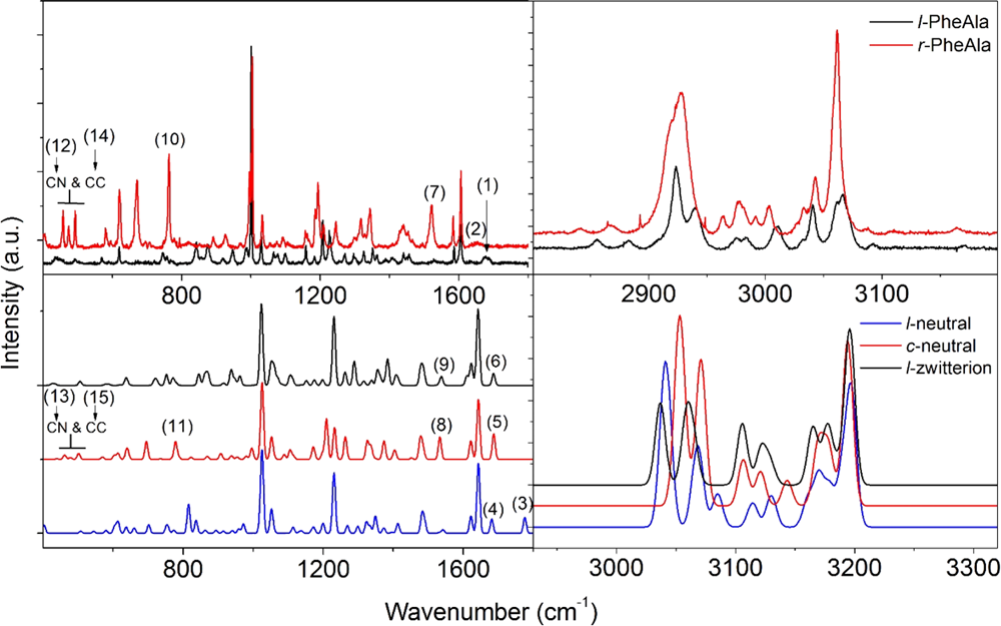
Top panel: comparison between the Raman spectra of *l*-PheAla (black curve) and *r*-PheAla (red curve) in the ranges of 400–1800 and 2800–3200 cm^–1^ on the left and right panels, respectively. Bottom panel: comparison between the simulated Raman spectra of the *l*-neutral (blue), *c*-neutral (red), and *l*-zwitterion (black) PheAla in the ranges of 400–1800 and 2900–3300 cm^–1^ on the left and right panels, respectively. The numerical labels refer to some of the main experimental and theoretical characteristic peaks of the different species in the region of 400–1800 cm^–1^ indicated in Table 2. We chose to show the simulated spectra in the region of 2930–3330 cm^–1^ of the CH vibrations (fully described in the SI) shifted by 130 cm^–1^ to better compare them with the experimental results. This blueshift in the high-frequency region of the simulated spectra is due to the lack of anharmonic contributions and intermolecular H-bonds (see the text, Results).

The experimental Raman spectra of *l*-PheAla and *r*-PheAla are shown in the upper panels of Figure 6, with the region of the CH stretching modes (>2800 cm^–1^) reported in the right-hand side. As in the case of the IR measurements, a few differences between these two samples are clearly recognizable. In the *r*-PheAla Raman spectrum, (i) new intense features appear as a triplet of peaks in the range of 400–500 cm^–1^ and two intense peaks around 760 and 1520 cm^–1^, while (ii) the features at about 770 and 1540 cm^–1^ decrease in intensity. In the region of CH stretching modes, there is a close similarity between the spectra, supporting the hypothesis that the structural rearrangement affects only minimally the hydrocarbon part of the molecule.

## Discussion

The experimental results introduced in the previous section suggest that above 65 °C, *l*-PheAla undergoes first a steady loss of water molecules trapped in the sample (*m*/*z* 18 and 17 in the mass spectra) and then a sudden burst of water molecules at 85 °C (Figures 2 and 3a). The latter is compatible with a bulk cyclization of the linear dipeptide, accompanied by the release of water due to the formation of an intramolecular peptide bond when the linear molecule “closes on itself”, rearranging into a cyclo structure. A further increase in temperature at 130 °C induces the desorption of the “newly formed” cyclo dipeptide (*m*/*z* 218), instead of the linear one (*m*/*z* 236), which was never observed through the whole temperature range of mass spectra acquisition.

To find consistent evidence of this hypothesis, we analyzed in detail the major changes in the IR spectral bands of *r*-PheAla and *l*-PheAla, with support of theoretical calculations. In the calculations, the linear neutral (*l*-neu), linear zwitterion (*l*-zwi), and cyclo neutral (*c*-neu) structures of PheAla, embedded in an implicit dielectric environment to enhance the similarities with the measured solid-state samples,^47^ have been considered, as reported in the inset of Figure 5. For the sake of clarity, we use *l*-zwi, *l*-neu, and *c*-neu labels, which all refer to the different PheAla species. The comparison reported in the SI between cyclic PheAla and pristine cyclic GlyPhe and AlaGly species, where some confusion can arise, is made using more precise labels such as *c*-neu(GlyPhe).

As a preliminary remark, we note that in *l*-PheAla, the dipeptide is found as a zwitterion or an inner salt, stabilized by the strong H-bond interaction between −NH_3_^+^ and −COO^–^ groups, also involving the central −C=O–NH– backbone, as well as by the presence of crystallized water molecules.^32^ However, zwitterions are not stable as isolated molecules in the gas phase and are readily converted into a neutral linear molecule. This same process may happen also on the surface of a solid sample, when heated, and it can be considered as a preliminary step of cyclization,^38^ even in the bulk, as discussed below. On the other hand, *c*-PheAla is an educated guess as a result of a thermal process that eliminates water and makes the parent ion disappear from the measured mass spectrum. For these reasons, our theoretical investigation has been extended to the three species *l*-neutral, *l*-zwitterion, and *c*-neutral. A comprehensive theoretical assignment of IR bands is listed in the SI, while the key features will be discussed here.

A comparison among the three simulated IR spectra and the experimental ones is reported in Figure 5. At first glance, there is a strong similarity between the measured spectrum of *l*-PheAla and the *l*-zwitterion simulation, as well as between that of *r*-PheAla and the *c*-neutral simulation. Regarding the region of high-frequency bands involving the C–H, N–H, and O–H stretching modes, the general blueshift of all calculated bands is due to two concurring effects, namely, the lack of anharmonic contributions and of intermolecular H-bonds.^48−51^ The latter is particularly significant in the case of NH- and OH-containing groups while barely affecting CH-containing groups. Even with these limits, the simulated spectra contain a broad contribution in the region of 2800–3200 cm^–1^ assigned to CH stretching modes, consistently observed in the experimental results (*cf.* the same region of the Raman spectra) as unperturbed by thermal treatment of the sample. On the other hand, the NH contribution appears as a doublet in the simulated *l*-zwitterion (3440 and 3510 cm^–1^) and in the measurement of *l*-PheAla (≈3300 and 3346 cm^–1^) and as a singlet in the simulated *c*-neutral (3570 cm^–1^) and the measured *r*-PheAla (3315 cm^–1^). The OH band at 3610 cm^–1^ suggests the presence of trapped water in *l*-PheAla, rather than the −COOH group of the simulated *l*-neutral molecule, as discussed in more detail below. Particularly relevant to the present discussion is the C=O stretching region, which is primarily affected by the formation of the second intramolecular peptide bond. The spectrum of *r*-PheAla is indeed characterized by a strong band at ≈1660 cm^–1^, matching well the one assigned to the asymmetric stretching of the two C=O bonds at 1684 cm^–1^ in the simulated spectrum of the *c*-neutral. Unfortunately, the *c*-PheAla sample is not available commercially, making a direct comparison of our TOF-MS and IR spectra impossible. However, measurements and simulations of IR spectra of the most closely resembling samples commercially available, *i.e.*, *c*-AlaGly and *c*-GlyPhe, show striking similarities with *r*-PheAla. As shown in Figure S2 (SI), the simulated IR spectra of these cyclic dipeptides are characterized by the same fingerprints of those simulated for *c*-neutral PheAla and measured for *r*-PheAla.

Regarding the *l*-PheAla IR spectrum, the weak contribution at 1676 cm^–1^ corresponds to the C=O stretching of the NH–C=O group in the *l*-zwitterion, while the broader peak at a lower wavenumber contains (among others) a strong contribution assigned to the asymmetric stretching of the COO^–^ group. Therefore, the *l*-neutral configuration of the molecule seems to be definitely ruled out, having the contribution of the −COOH group falling at 1776 cm^–1^, *i.e.*, well above the NH–C=O group (1681 cm^–1^) and unmatched by any measured line. The frequencies and assignments discussed above are reported in Table 2 (2.1). Further analysis of the region between 1600 and 1250 cm^–1^ confirms the identification of the *l*-PheAla and *r*-PheAla samples with the *l*-zwi and *c*-neu structures, respectively, as detailed in Figure S2.

**Table 2:** Experimental and Theoretical Frequencies Together with the Proposed Assignment of Some of the IR (2.1) and Raman (2.2) Bands Identifying the Main Changes in the Vibrational Modes of the PheAla Molecule Compatible with a Cyclization Process (See the Text)^a^

(2.1) Infrared Cyclization Fingerprints					
assignment	experimental frequencies (cm^–1^)		theoretical frequencies (cm^–1^)		
	*l*-PheAla	*r*-PheAla	*l*-neutral	*c*-neutral	*l*-zwitterion
OH	3610 (1)	absent	3721 (2)	absent	absent
NH	3346 (3)	3315 (5)	3000–3200	3570 (6)	3510 (7)
	3300 (4)				3440 (8)
CH (phenyl)	3025–3100 (9)	3025–3100	3150–3200 (10)	3150–3200	3150–3200
CH (alkyl)	2800–3025 (9)	2800–3025	2900–3150 (10)	2900–3150	2900–3150
CO	1676 (C=O)	1660 (C=O) (11)	1776 (−COOH)	1684 (C=O) (12)	1686 (C=O)
	≈1600 (COO^–^) (13)		1681 (NH–C=O)		1614 (COO^–^) (14)

(2.2) Raman Cyclization Fingerprints					
assignment	experimental frequencies (cm^–1^)		theoretical frequencies (cm^–1^)		
	*l*-PheAla	*r*-PheAla	*l*-neutral	*c*-neutral	*l*-zwitterion
CO	1680 (C=O) (1)	≈1650 (C=O) (2)	1775 (−COOH) (3)	1687 (C=O) (5)	1686 (C=O) (6)
			1681 (NH–C=O) (4)		
CC and CN (DKP)	absent	1520 (7)	absent	1533 (8)	absent
NH	1527	absent	1540	absent	1538 (9)
complex skeletal mode (phenyl and DKP)	757 (10)	763	773	778 (11)	770
CN (DKP)	absent	458; 474 (12)	absent	462; 478 (13)	absent
CC (phenyl and DKP)	492	493 (14)	508	502 (15)	506

a The numerical labels refer to the characteristic peaks in the IR and Raman spectra of Figures 5 and 6. The frequencies of the main vibrational bands have been assigned according to refs (52−59) and present theoretical simulations.

The analysis of vibrational IR bands in *l*-PheAla and *r*-PheAla, consistent with the predictions for *l*-zwi and *c*-neu structures, respectively, confirmed that a structural rearrangement occurred in the solid phase due to the thermal treatment. This structural rearrangement is fully compatible with a temperature-driven cyclization in the condensed phase. This result is also consistent with the cyclo peptide formation previously reported^13^ in the temperature range of 170–215 °C and with the mass spectra of dipeptides.^14^

Further support to this conclusion is provided by the comparison of Raman spectra measured on the *l*-PheAla and *r*-PheAla samples, shown in Figure 6 and completely assigned in the SI (Tables S1–S3) through the comparison with simulations. In these spectra, the characteristic Raman lines of the [phenyl–CH_2_]– group at 1605, 1206, 1029, and 1001 cm^–1^ have been assigned by means of a close comparison between the two PheAla measurements in Figure 6 and the Raman spectra of *c*-GlyPhe (containing [phenyl–CH_2_]) and *c*-AlaGly (not containing this group), which are shown in the SI (Figure S5 and Table S4). The simulations of the Raman spectrum also offer the possibility to unequivocally assign the contribution listed in the following. The strongest contribution at 1001 cm^–1^, accompanied by a weaker satellite at 1029 cm^–1^, is assigned to a CC stretching of the phenyl ring (1026 and 1052 cm^–1^ in the case of *c*-neu simulation; 1023 and 1051 cm^–1^ in the case of *l*-zwi simulation). Weaker contributions around 1605 and 1200 cm^–1^ are assigned to a different CC stretching of the phenyl ring (contributions at 1621 and 1643 cm^–1^ in the case of the *c*-neu structure; 1623 and 1643 in the case of the *l*-zwi structure) and to more localized vibrations involving the −CH_2_– moiety connected to the phenyl ring (contributions at 1209, 1232, and 1263 cm^–1^ in the case of *c*-neu; a single, stronger contribution at 1231 cm^–1^ in the case of *l*-zwi). We remark the fact that these experimental bands are insensitive to the thermal treatment and can therefore be safely assigned to the side [phenyl–CH_2_]– group, practically unaffected by cyclization. Once assigned the precise fingerprint of [phenyl–CH_2_], we can analyze a few specific differences between the *l*-PheAla and *r*-PheAla spectra (Table 2 (2.2)), referring the interested reader to the SI for a complete assignment of the Raman spectra.^54,59−62^ In *l*-PheAla, the broad and weak band at ≈1680 cm^–1^ is compatible with the band computed at 1686 cm^–1^ in the *l*-zwi configuration, assigned to the C=O stretching of the CO–NH group, also involving the NH_3_^+^ group that, interacting with C=O through a H-bond,^63^ may cause an overestimation of the vibrational frequency in this model system. On the other hand, the weak feature at 1650 cm^–1^ in *r*-PheAla is compatible with the band calculated at 1687 cm^–1^ in the *c*-neu configuration and assigned to the symmetric stretching of the two C=O bonds.

We further focus on three selected regions of the spectrum in Figure 6 characterized by marked differences between the two samples, *l*-PheAla and *r*-PheAla.

The band around 1520 cm^–1^ in *r*-PheAla matches with a strong band at 1533 cm^–1^ in the *c*-neu calculation, assigned to a peculiar stretching of CC and CN bonds in the DKP ring, while the tiny feature in the same region of *l*-PheAla is assigned to a localized bending mode of NH in the *l*-zwi calculation.

The strong band at 763 cm^–1^ in *r*-PheAla is assigned to a complex skeletal mode involving both phenyl and DKP rings at 778 cm^–1^ in the *c*-neu simulation, while the weak, complex band in the same region of *l*-PheAla is assigned to the bending modes of C=O and COO^–^ at 752 and 771 cm^–1^, respectively, in the *l*-zwi simulation.

Finally, the triplet band in the 400–500 cm^–1^ region of *r*-PheAla is assigned to the CN bending of the DKP ring (462 and 478 cm^–1^) and to the out-of-plane CC bending of the phenyl ring (502 cm^–1^). Only this last contribution survives in the *l*-PheAla spectrum, assigned to the same phenyl mode at 506 cm^–1^ in the *l*-zwi calculation.

As anticipated in the previous section, the region of 2800–3200 cm^–1^, rich in several well-resolved bands, is very descriptive of the different CH stretching modes in the molecule. However, these features barely change between linear and cyclo structures: the region above 3030 cm^–1^ (above 3150 cm^–1^ in Raman simulations) is assigned to phenyl stretching modes, while the region below is assigned to methyl, methylene, and methyne stretching modes. A complete assignment of these bands in the calculated spectra of *c*-neu and *l*-zwi configurations is also reported in the SI (Figure S4).

As in the case of IR spectra, to confirm that the features observed in *r*-PheAla are fully compatible with those observed for similar cyclo species, the Raman spectra of the pristine *c*-GlyPhe and *c*-AlaGly samples have been measured and compared with the simulated *c*-neu(GlyPhe) and *c*-neu(AlaGly) spectra. The corresponding results, reported in the SI, support once again the hypothesis that *l*-PheAla has undergone a cyclization process.

After this fine spectroscopic comparison, we can reinterpret TGA results on more solid ground: the mass loss of ≈(6 ± 2)% observed at 120 °C in the TGA is consistent with the mass ratio of the water molecule and *l*-PheAla (7.6%) expected for a 100% efficient “intramolecular” peptide bond formation, which releases a water molecule for each linear dipeptide, which undergoes cyclization. It follows that in the TOF-MS measurements, the surge of the water peak at about 85 °C corresponds to the water release due to peptide bond formation, which then results in the appearance of the *m*/*z* 218 (*c*-PheAla parent ion), while there is no trace of the parent ion of the *l*-PheAla species (*m*/*z* 236). As opposed to previous works on mass spectra of dipeptides,^14−16^ in this specific dipeptide and experimental conditions, the cyclization process involves ≈100% of the sample, and we never observed the *l*-PheAla parent ion.

Once established that the cyclization of *l*-PheAla occurs in the condensed phase, we now focus on the mechanism leading to such cyclization. Let us reconsider first the spectroscopic results discussed in detail above. Starting from about 65 °C, we observe water released from the pristine sample, up to the substantial burst at 85 °C. Interstitial water stabilizes the zwitterionic crystal structure of PheAla, and its initial slow release favors a rearrangement in which linear neutral molecules may tend to coil, especially on the surface of the sample where the zwitterion is only partially screened by surrounding molecules and water molecules are steadily extracted from the sample. In this phase of the heating process, protons may even rearrange in the molecular network to form neutral linear peptides. However, the lack of any fingerprint of *l*-neutral molecules in the IR and Raman spectra of both *l*-PheAla and *r*-PheAla samples indicates that such a configuration can be only an intermediate stage between 65 and 85 °C, preceding the actual cyclization. The large production of water molecules at 85 °C indicates a bulk process compatible with the cyclization of the majority or even all the molecules.

Building on this ground, the cyclization has been further investigated by simulations based on density functional theory. We note that the description of a reaction coordinate involving a dipeptide in a solid-state environment, characterized by strong, anisotropic H-bonds between molecules, and also involving water molecules as possible players is out of reach for accurate, quantitative calculations. Our forced choice has been to model the process by considering a single molecule embedded in an isotropic dielectric environment, which can stabilize the initial zwitterion configuration. The significant effect of water molecules on the process has been taken into account by calculating reaction barriers in the presence or absence of a single water molecule.

Recently, Li *et al.*(38) investigated the formation of symmetric cyclo dipeptides in water solution from aliphatic amino acids, using density functional theory simulations. In detail, the reported results individuated three reaction pathways for the cyclization process of a linear dipeptide. Two fully intramolecular reactions differ by the formation of a transition state involving a four-membered C–O–H–N ring (path 1) or of a locally stable CH_2_–C(OH)_2_–NH intermediate (path 2). A third, intermolecular path (path 3 below) is characterized instead by the active participation of a single water molecule, serving as a bridge for proton transfers through the formation of transition states involving six-membered rings, separated by the same locally stable CH_2_–C(OH)_2_–NH intermediate of path 2.

**Figure 7:**
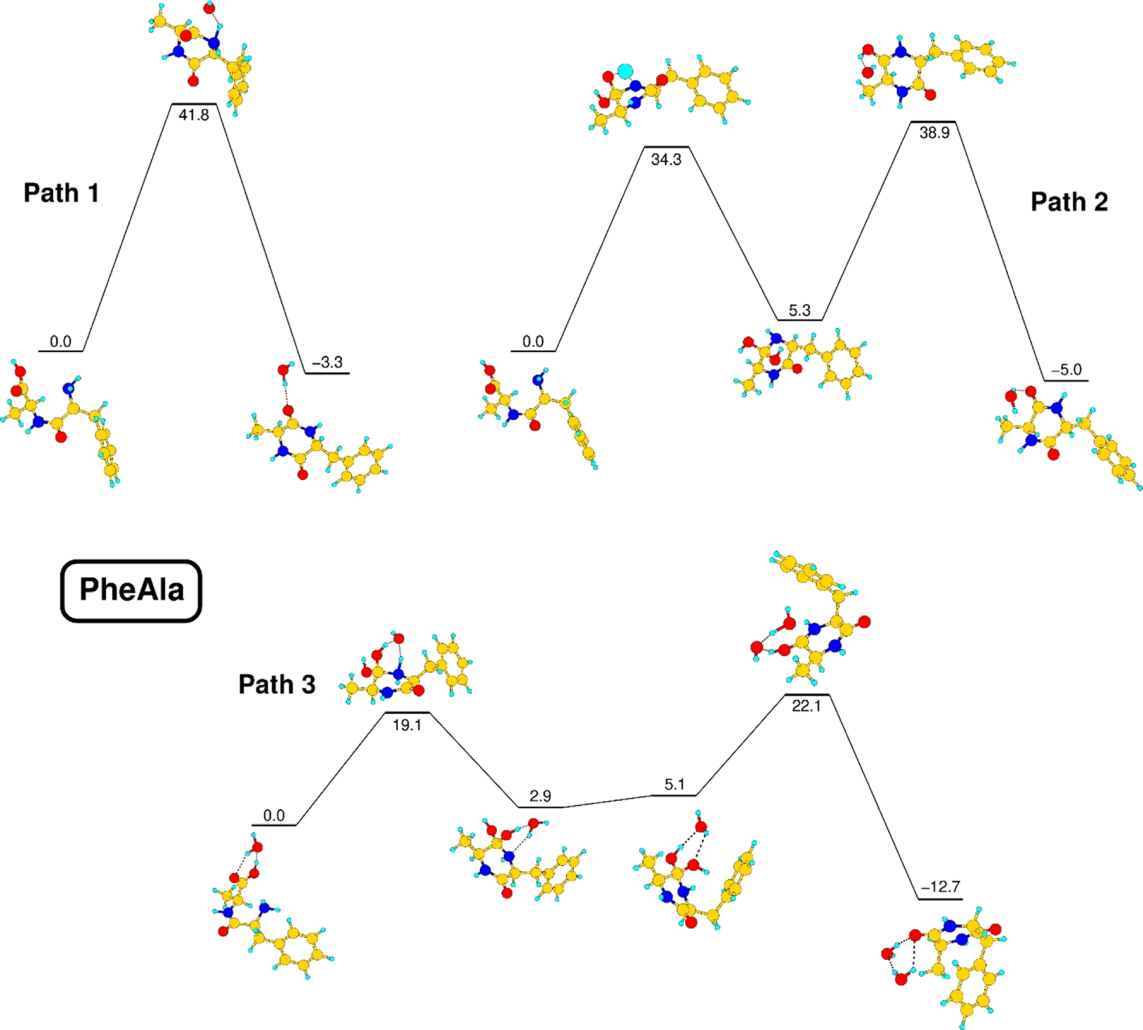
Optimized structures of the stationary points of intramolecular (paths 1 and 2) and intermolecular (path 3) cyclization reactions of *l*-PheAla. Total energy values, calculated in an M06-2X@def2-TZVPP framework, are expressed in kcal/mol. See the text.

Our computational approach has been first tested in the case of the cyclization of the GlyGly dipeptide, yielding results consistent with those previously obtained in ref (38), as discussed in the SI. The same approach has then been applied to the *l*-PheAla dipeptide, and the results for the three paths are shown in Figure 7.

The results show that in our simplified model, there is a small but not negligible energy gain of 2.4 kcal/mol in the transfer of one proton from NH_3_^+^ to COO^–^, with a small barrier of 0.1 kcal/mol (the path is not shown in Figure 7 as it is a preliminary step for all paths shown there), confirming the likely spontaneous conversion of the zwitterion in a neutral molecule outside of strongly H-bonded environments. Intramolecular reactions are characterized by high barriers in our model, with values of 34.3 kcal/mol in the case of path 2 and up to 41.8 kcal/mol in the case of path 1. Even if these barriers can be considered as upper limits and may be lowered when the molecule is in contact with a dipeptide substrate, they cannot be considered as highly compatible with a thermal process activated at 85 °C. The insertion of a single active water molecule may appear as a small perturbation of the system, but it is of great significance for the understanding of the cyclization process. Such a water molecule participates in the formation of six-membered rings as reaction intermediates, which are more stable than the four-membered rings of path 2. Moreover, water promotes proton transfer behaving as an acceptor–donor bridge, a role that cannot be played in a single step by surrounding peptides. The presence of a single water molecule is therefore able to lower the reaction barrier of the limiting step down to 19.1 kcal/mol. This peculiar role of water molecules nicely fits in the observation of a rearrangement process involving bulk molecules at 85 °C. The increased availability of water molecules, which are able to significantly lower the cyclization barrier, may suddenly induce a chain reaction, as each cyclization reaction inserts one further water molecule into the system, which can explain the results of TG-DTA and mass spectrometry measurements.

We stress the fact that the reaction pathways investigated in the case of our simplified model may be directly applied to an isolated *l*-PheAla molecule only. A more realistic cyclization mechanism in the condensed phase must be surely considered as a much more complex process, involving adjacent molecules likely favoring the neutralization of the zwitterions on the surface of the powder. A chain reaction process in which the release of water molecules after the cyclization may enhance the efficiency of the process for other *l*-dipeptides cannot be excluded. In this regard, preliminary results, briefly discussed in the SI, suggest that cooperative effects involving the interaction between *l*-PheAla molecules in the solid state may alter the shape of the minimum-energy path along the reaction coordinate, lowering the corresponding barriers.

## Conclusions

A combined experimental and theoretical study has been carried out to shed light on the thermally induced cyclization of the *l*-PheAla dipeptide. Measurements of mass spectra *vs* temperature and TG-DTA provided evidence of a sudden burst of water desorption around 85 °C in UHV. Moreover, the *l*-PheAla parent ion has never been detected, while at a temperature of about 130 °C, the *m*/*z* of the *c*-PheAla parent ion (*m*/*z* 218) and other fragments attributable to its fragmentation have been observed. The comparison of the measured IR and Raman spectra of the pristine *l*-PheAla and *r*-PheAla samples with the simulated spectra of the *l*-zwitterion, *l*-neutral, and *c*-neutral PheAla structures allowed us to establish that the pristine sample is in its zwitterionic form and that all the main spectroscopic features of *r*-PheAla are fully compatible with those typical of a cyclo dipeptide species. Such a compatibility is confirmed by further comparison with the IR and Raman spectra of commercially available, very similar compounds like *c*-GlyPhe and *c*-AlaGly. All these pieces of evidence lead to the sound statement that a thermally induced cyclization occurs before sublimation of *l*-PheAla in vacuum, *i.e.*, a massive process involving the bulk occurs in the condensed phase. A theoretical investigation of the cyclization reaction mechanism indicates that even a single water molecule plays an essential catalytic role to lower reaction barriers, which are otherwise too high to be compatible with a thermally induced cyclization process. Preliminary results also suggest a complementary role of cooperative peptide–peptide and water–peptide intermolecular interactions in the further lowering of the energy barrier. Since the investigated mechanism does not require the presence of activating agents or chemical precursors but only a single water molecule to halve the potential energy barrier, it provides an effective strategy to protect peptides, converting them from the fragile, by comparison, *l*-structure into the more robust and stable *c*-structure, thus preserving the amino acid sequence from further decomposition in harsh environments. Hence, if the *l*-PheAla dipeptide can be generated abiotically by coupling the two constituent amino acids, then this cyclization process may occur spontaneously on interstellar objects like comets and carbonaceous chondrites experiencing thermal alterations during their travel in space, as well as in the early Earth after the interaction with the primordial terrestrial environment.

Finally, the cyclization of *l*-dipeptides in the solid phase may lead to the formation of peculiar self-assembled 2D nanostructures, while usually, the linear molecules tend to form amorphous films under the same conditions. In light of further development of innovative techniques for the preparation of nanomaterials based on oligopeptides, the ability to handle thermally induced cyclization processes is useful to control the properties of such nanomaterials via the growth of specific nanostructures triggering different self-assembly mechanisms, corresponding to different starting peptides.

## Supporting Information Available

Extended description of the *l*-PheAla TG-DTA results and Raman and IR measurements performed on *c*-GlyPhe and *c*-AlaGly; complete IR and Raman theoretical frequencies of *l*-neu, *l*-zwi, and *c*-neu PheAla molecules and *c*-neu GlyPhe and AlaGly molecules; cyclization mechanism in water solution of the linear GlyGly dipeptide and theoretical preliminary results on the *l*-PheAla cyclization mechanism in the solid state (PDF)
