# Supplementary material for: Insights into the Thermally Activated Cyclization Mechanism in a Linear Phenylalanine-Alanine Dipeptide
Source: J Phys Chem B. 2022 Apr 19;126(16):2968–78. doi: 10.1021/acs.jpcb.1c10736 (PMC9059117; doi:10.1021/acs.jpcb.1c10736)
Supplement: Supplementary file 1 — jp1c10736_si_001.pdf [file jp1c10736_si_001.pdf]

Insights in Thermally Activated Cyclization Mechanism in Linear Phenylalanine-Alanine Dipeptide

Laura Carlini[1*], Jacopo Chiarinelli[1], Giuseppe Mattioli[1], Mattea Carmen Castrovilli[1], Veronica Valentini[1], Adriana De Stefanis[1], Elvira Maria Bauer[1], Paola Bolognesi[1], Lorenzo Avaldi[1]

[1]CNR-Istituto di Struttura della Materia (CNR-ISM), Area della Ricerca di Roma 1, Monterotondo Scalo, Italy

The supplementary material includes an extended description of the L-Phenylalanyl-L-Alanine (namely *l*-PheAla) TG-DTA results, the Raman and IR measurements performed on (3S)-3-Benzyl-2,5-piperazinedione (*c*-GlyPhe) and (3S)-3-Methyl-2,5-piperazinedione (*c*-AlaGly). Complete IR and Raman theoretical frequencies of linear neutral (*l*-neu), linear zwitterion (*l*-zwi) and cyclo neutral (*c*-neu) structures of the isolated PheAla molecule and *c*-neu structures of GlyPhe and AlaGly molecules are reported. The cyclization mechanism in water solution of linear GlyGly dipeptide using simulations based on density functional theory is also discussed. Finally, a brief discussion of the theoretical preliminary results on the *l*-PheAla cyclization mechanism in the solid state is reported.

1. Thermogravimetric Analysis

The Thermogravimetric and Differential Thermal Analysis (TG-DTA) measurements performed on the *l*-PheAla sample over the temperature range from room temperature up to 800 °C are shown in Figure S1. The DTA results show four endothermic enthalpy peaks (measured as heat flow in mW) centered at ≈ 105, 135, 260 and 362 °C, respectively. The mass losses (%) and the related errors, as well as the temperature ranges (reported in Figure S1) have been estimated by a deconvolution method applied on the derivative of the weight curve. Through this operation, five different steps of mass loss have been identified. The fitting procedure, performed using Voigt functions, indicates that the first two steps of mass loss overlap, and correspond to a total weight loss of about 16%. Also the last two steps are partially overlapped and correspond to the higher contributions to the mass loss, 31% and 40%, with onset points at ≈ 300 and 314 °C, respectively.

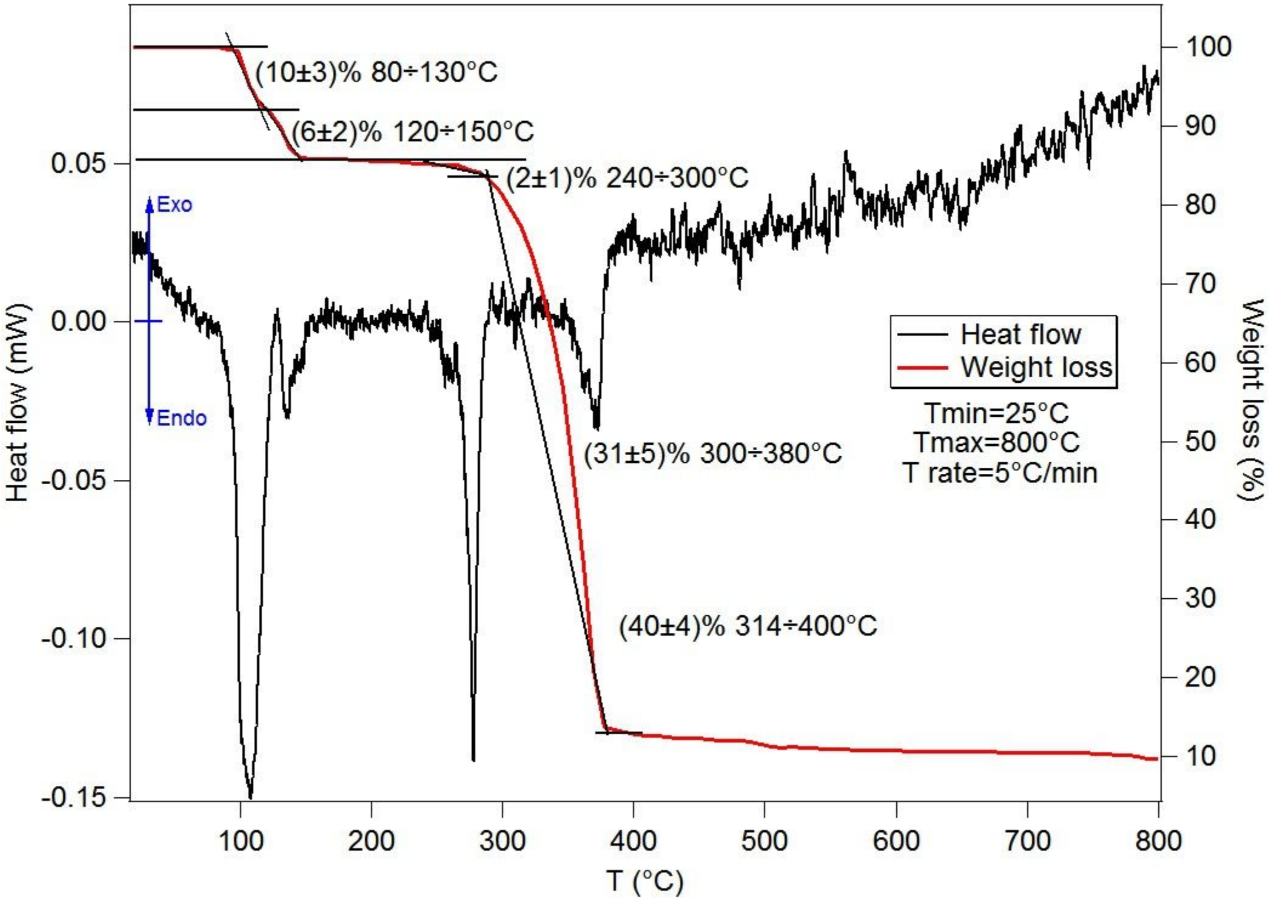

Figure S1. *TG-DTA measurements of the l-PheAla sample. The weight losses (%) and their related errors and temperature ranges are reported.*

More in detail, the first endothermic peak in the temperature range 80-130 °C is related to a first weight loss of about 10%. The second step of mass loss (≈ 6%) with onset point at 120 °C partially overlaps with the first one and is accompanied by another endothermic enthalpy peak. Further heating the sample (above 200 °C) leads to the appearance of a third prominent endothermic peak (onset point ≈ 240 °C) associated to a small step of mass loss of (2±1)%. In the temperature range 300-380 °C there is a dramatic mass decrease of ≈ 31%, without noticeable enthalpy changes. Finally, partially overlapped with the previous one, a 40% weight loss is observed between 314 and 400 °C, in concomitance with the last endothermic peak. The presence of two main steps of mass loss suggests that the residual sample may undergo a "two steps sublimation" between 270 and 400 °C with a total weight loss of 71%.

On the ground of the hypothesis of a cyclization mechanism of the *l*-PheAla molecule in the condensed phase[1-4], we propose the following interpretation of the TG-DTA results. The mass loss of ≈ (6±2)% observed at 120 °C may be consistent with the mass ratio of water molecule and *l*-PheAla of 7.6% expected for a 100% efficient 'intramolecular' water-emission process. The cyclization hypothesis may also explain the two steps of mass loss above 300 °C. Assuming the formation of the cyclo species (*c*-PheAla, 218 amu), one may suppose that the most probable dissociation

mechanism of this species involves the loss of the phenyl ring with the formation of two main fragments with m = 91 (benzyl radical) and 127 amu. The mass ratio of 0.42 for m = 91 amu is in good agreement with the 0.44 ratio of the first weight loss (31%) with respect to the total mass loss of 71% in the range 300-400 °C. For the last step (40%) the ratio is equal to 0.56 which is consistent with 0.58 mass ratio of m = 127 amu with respect to the *c*-PheAla molecule (218 amu). The last endothermic peak related to this step could be due to another structural rearrangement during the "two steps sublimation" of the residual sample.

2. IR and Raman results

The comprehensive theoretical assignments of the IR and Raman spectra calculated on the optimized structures of the linear neutral (*l*-neu), cyclo neutral (*c*-neu) and linear zwitterion (*l*-zwi) PheAla molecule are reported in Table S1, S2 and S3, respectively.

***l*-neu(PheAla) normal modes analysis**				
**Normal modes**			**IR activity (a.u.)**	**Raman activity (a.u.)**
**Mode**	**Wavenumber (cm^{-1})**	**Assignment**		
7:	32,89		4,30507	2,81961
8:	39,48		3,22151	0,88711
9:	52,72		1,59956	12,46752
10:	69,09		3,3174	1,25256
11:	88,44		3,26152	2,50555
12:	112,39		5,06723	0,82693
13:	122,24		1,66532	8,02069
14:	186,99		69,22102	2,09002
15:	209,80		7,40331	5,49028
16:	216,64		2,56251	4,87692
17:	237,20		0,73065	0,38836
18:	286,75		6,8916	1,27885
19:	294,42		3,81838	2,24954
20:	314,62		32,06456	1,28197
21:	343,51		8,70752	0,68874
22:	354,38		18,65213	3,61701
23:	362,86		29,99008	2,5499
24:	403,24	complex	16,64792	11,82621
25:	417,38	dCC (phenyl)	0,13167	0,25039
26:	507,71	complex	47,14025	3,03037
27:	544,53	complex	46,06644	2,91508
28:	579,73	dOH	161,17901	4,2498
29:	604,66	dNH	48,85808	10,37847
30:	614,15	dNH	53,55707	16,89617
31:	637,51	dCC (phenyl)	0,98325	9,1728
32:	660,34	complex	18,53256	7,75362
33:	701,79	complex	35,65706	12,2362
34:	716,55	dCH (phenyl)	103,49238	0,19241
35:	752,24	complex	17,24377	7,99968

36:	756,96	d(COOH)	30,23749	6,65419
37:	773,29	complex	32,36432	4,52056
38:	815,57	vCC (C2+C5)	9,98874	41,792
39:	837,15	vCC (C13+C14)	4,25211	18,84906
40:	862,99	dCH (phenyl)	0,22861	4,4087
41:	893,84	complex	3,04605	4,19885
42:	914,21	dNH2	250,81156	3,14819
43:	940,44	dCH (phenyl)	8,43461	3,99264
44:	958,84	dCH(methyne C5 + methyl C29)	0,89596	6,49354
45:	972,33	vCC (C9+C13)	12,74034	13,48856
46:	1000,13	dCH (phenyl)	0,39127	0,31255
47:	1017,25	dCH (phenyl)	9,37556	4,20049
48:	1021,68	complex	26,86908	6,99454
49:	1025,43	dCC (phenyl)	1,76288	115,2903
50:	1052,3	dCC (phenyl)	8,72645	34,53923
51:	1059,25	dCH(methyne C5 + methyl C29)	36,54003	3,29464
52:	1097,01	complex	25,05148	0,66983
53:	1113,80	vCC (C5+C29)	50,12281	8,84453
54:	1130,38	vCN+vCO(C9+N10,C2+O3)	389,57754	1,4098
55:	1137,71	vCN+vCO(C9+N10,C2+O3)	115,48171	3,51102
56:	1166,60	complex	7,04936	3,15984
57:	1173,15	dCH (phenyl)	0,11935	7,94377
58:	1199,09	vCN (C5+N6)	16,44606	4,54909
59:	1199,56	dCH (phenyl)	3,96985	10,25402
60:	1227,78	complex	0,99452	8,83722
61:	1230,92	vCC (C13+C14)	2,43196	80,75598
62:	1269,08	dNH	107,99107	11,268
63:	1298,77	dOH+dCH (methyne C5)	10,08897	10,19507
64:	1323,34	vCC (phenyl)	4,10707	16,68461
65:	1333,82	complex	11,19143	10,14829
66:	1348,84	complex	0,90746	24,17256
67:	1354,79	complex	6,84017	1,60903
68:	1373,08	dCH (phenyl)	0,01258	7,82895
69:	1396,17	dCH (methyne C5 + methyl C29)	12,72862	1,75845
70:	1412,34	dCH (methyne C9)	5,66672	7,89059
71:	1414,23	dCH (methyne C5 + methyl C29)	55,74187	6,72991
72:	1479,53	dCH (methylene C13)	5,32191	4,79903
73:	1483,21	dCH (methyl C29)	8,30933	25,19979
74:	1488,39	vCC+dCH (phenyl + methylene C13)	16,27977	3,18527
75:	1490,91	dCH (methyl C29)	24,79807	10,68774
76:	1531,34	vCC (phenyl)	26,77726	1,43205
77:	1542,21	dNH	347,98043	5,36748
78:	1621,80	vCC (phenyl)	1,22503	24,88831
79:	1639,80	dNH2	28,76906	4,18478
80:	1643,22	vCC (phenyl)	13,79922	97,75923
81:	1681,43	vC=O (-CO-NH-)	620,2367	20,5536
82:	1775,66	vC=O (-COOH)	511,24699	23,13132
83:	3039,46	vCH (methylene C13)	39,31953	496,14555

84:	3043,24	vCH (methyl C29)	18,17682	432,53903
85:	3066,25	vCH (methylene C13 + methyne C9)	18,11752	225,09888
86:	3069,9	vCH (methyne C5)	13,43647	228,13596
87:	3084,87	vCH (methylene C13 + methyne C9)	36,57964	167,78798
88:	3114,32	vCH (methyl C29)	23,16386	122,68801
89:	3130,00	vCH (methyl C29)	18,63484	158,86127
90:	3160,67	vCH (phenyl)	9,14078	142,03514
91:	3169,83	vCH (phenyl)	0,45995	256,6803
92:	3178,97	vCH (phenyl)	27,45376	196,9221
93:	3189,66	vCH (phenyl)	32,35672	276,15731
94:	3197,41	vCH (phenyl)	8,41027	673,03176
95:	3482,14	vNH_2	4,10462	309,61038
96:	3548,91	vNH_2	9,06559	146,81682
97:	3619,66	vNH	75,33419	94,14817
98:	3721,08	vOH	163,40223	216,63152

Table S1. *Proposed assignment and theoretical frequencies of IR and Raman normal modes calculated for the isolated l-neu(PheAla) molecule using the B3LYP functional. On the right side IR and Raman activities are reported, where IR activity stands for the squared modulus of the transition dipole moment.*

***c*-neu(PheAla) normal modes analysis**				
**Normal modes**			**IR activity (a.u.)**	**Raman activity (a.u.)**
**Mode**	**Wavenumber (cm^{-1})**	**Assignment**		
6:	47,76		0,4257	3,2265
7:	61,05		1,41439	11,22101
8:	73,18		0,78336	5,66269
9:	86,44		10,29816	1,60507
10:	112,04		1,91065	1,2741
11:	119,72		0,39452	6,49413
12:	196,00		0,75254	5,04078
13:	231,39		0,29339	0,2932
14:	257,28		3,48869	4,10583
15:	269,68		1,33073	2,04735
16:	370,14		0,27141	0,45129
17:	386,85		7,85431	1,70659
18:	402,33	dCO	49,01888	0,46098
19:	417,70	dCC (phenyl)	0,02856	0,09272
20:	439,29	complex	8,87139	0,90332
21:	461,77	dCN (dkp ring)	4,10853	6,30006
22:	477,90	dCN (dkp ring)	8,25512	2,64644
23:	501,78	dCC (phenyl)	18,87832	8,65645
24:	569,69	dNH	33,91813	3,34367
25:	603,62	dNH	16,37407	5,72049
26:	614,29	dNH	112,06244	8,6057
27:	637,95	dCC (phenyl)	0,32638	8,4594
28:	641,98	dNH	95,68674	9,86608

29:	695,21	dNH	119,73327	25,96283
30:	719,96	dCH (phenyl)	93,10633	0,21175
31:	735,25	complex	31,47806	0,86949
32:	769,13	complex	19,15882	1,41966
33:	778,40	complex	11,74645	25,26784
34:	787,19	complex	10,48909	3,30739
35:	822,91	complex	27,80423	2,87264
36:	868,83	dCH (phenyl)	0,51227	4,13309
37:	907,69	complex	5,83406	8,65754
38:	938,07	dCH (methylene C9)	23,42446	5,07576
39:	954,16	dCH (phenyl)	5,48064	3,46984
40:	980,38	complex	28,86018	4,62813
41:	996,15	vCC (C8+C9)	3,72259	16,37991
42:	1004,32	dCH (phenyl)	0,31692	0,15517
43:	1022,48	dCH (phenyl)	0,46221	2,32925
44:	1025,55	vCC (phenyl)	0,06897	109,55182
45:	1052,37	vCC (phenyl)	9,09769	31,6931
46:	1058,69	complex	23,03561	3,57054
47:	1088,50	complex	13,13427	6,63788
48:	1105,45	vCC (C4+C25)	15,05004	13,59749
49:	1115,06	complex	59,42935	4,49322
50:	1170,83	complex	16,00871	11,01576
51:	1173,27	dCH (phenyl)	0,53793	7,72426
52:	1198,51	dCH (phenyl)	1,47024	9,47002
53:	1209,34	dCH (methylene C9 + methyne C8)	6,43692	58,35553
54:	1232,28	vCC (C9+C10)	9,85799	46,02489
55:	1263,07	dCH (methylene C9 + methyne C8)	5,86501	32,67247
56:	1323,43	dCH (methyne C4)	51,12647	6,57418
57:	1326,36	dCH (methyne C8)	37,53828	20,98721
58:	1336,18	dCH (methyne C4)	53,24457	19,08741
59:	1347,41	vCC (phenyl)	146,81135	1,50893
60:	1368,36	dCH (phenyl)	14,52565	2,44063
61:	1373,96	dCH (methylene C9 + methyne C8)	7,15599	25,53432
62:	1400,89	dCH (methyl C25)	6,21127	3,84697
63:	1405,03	dkp-ring	1,41374	10,91504
64:	1451,87	vCC+vCN	238,37127	2,0028
65:	1476,19	dCH (methylene C9)	76,55765	13,37354
66:	1478,41	dCH (methyl C25)	103,92147	16,47009
67:	1484,09	dCH (methyl C25)	15,03793	13,3293
68:	1487,09	complex	12,79194	2,98341
69:	1491,93	complex	35,74532	1,0025
70:	1530,85	dCH (phenyl)	15,94765	3,65069
71:	1533,33	vCC+vCN (dkp-ring)	3,61962	29,18903
72:	1621,50	vCC (phenyl)	1,20502	26,60324
73:	1643,08	vCC (phenyl)	5,22506	86,46856
74:	1683,81	vC=O	1697,55935	0,10413
75:	1686,93	vC=O	34,13251	36,75446
76:	3051,26	vCH (methyl C25)	27,91857	321,83868
77:	3053,02	vCH (methyne C4)	20,74164	487,95674

78:	3056,04	vCH (methylene C9)	22,9751	237,10587
79:	3070,74	vCH (methyne C8)	35,52159	745,8102
80:	3106,52	vCH (methylene C9)	16,18354	235,48498
81:	3120,91	vCH (methyl C25)	19,08083	175,30637
82:	3143,32	vCH (methyl C25)	8,67111	130,15409
83:	3166,26	vCH (phenyl)	4,58193	40,04566
84:	3169,81	vCH (phenyl)	0,54399	283,33375
85:	3177,89	vCH (phenyl)	16,01699	284,23049
86:	3184,51	vCH (phenyl)	42,61702	27,18362
87:	3194,25	vCH (phenyl)	14,60632	840,3919
88:	3569,68	vNH	139,66797	16,43993
89:	3570,54	vNH	2,97925	510,48376

Table S2. *Proposed assignment and theoretical frequencies of IR and Raman normal modes calculated for the isolated c-neu(PheAla) molecule using the B3LYP functional.*

***l*-zwi(PheAla) normal modes analysis**				
**Normal modes**			**IR activity (a.u.)**	**Raman activity (a.u.)**
**Mode**	**Wavenumber (cm^{-1})**	**Assignment**		
6:	23,67		1,47116	7,97606
7:	29,52		9,34001	0,92636
8:	44,81		3,67283	0,38289
9:	54,06		4,40732	1,95191
10:	61,87		1,18313	4,11791
11:	81,85		24,97686	6,19082
12:	105,09		13,87081	3,89946
13:	128,74		25,63537	0,43372
14:	168,50		19,18264	2,28503
15:	228,33		7,01927	1,08888
16:	233,45		10,09563	0,81195
17:	250,55		7,12744	3,21317
18:	252,48		31,62952	6,19153
19:	290,11		57,52741	2,30222
20:	307,57		20,37984	3,41788
21:	344,55		49,77235	0,67826
22:	362,96		9,30669	0,19903
23:	417,70	dCC (phenyl)	0,20766	0,06133
24:	425,76	complex	6,92986	2,34561
25:	434,46	complex	21,23931	2,02323
26:	505,79	dCC (phenyl)	30,29617	5,74271
27:	578,82	complex	0,65059	1,72765
28:	587,35	complex	12,8255	1,612
29:	622,37	dNH	102,55032	1,1704
30:	636,92	dCC (phenyl)	1,00398	9,73666
31:	642,03	complex	42,37576	2,16705
32:	718,04	complex	9,06936	6,78788
33:	724,62	complex	101,85711	6,56524
34:	752,48	dCO	1,3255	15,56073

35:	770,90	dCOO	42,2254	10,59281
36:	780,04	complex	11,53257	3,17825
37:	845,01	complex	27,66134	16,47854
38:	861,49	complex	8,06883	13,06981
39:	867,75	dCH (phenyl)	3,41542	8,82383
40:	872,00	complex	18,20757	11,52541
41:	915,65	complex	19,92326	3,2007
42:	937,23	complex	26,41279	22,3664
43:	947,85	dCH (phenyl)	35,93649	8,82202
44:	962,21	vCC (C10+C26)	92,66744	13,45855
45:	1004,80	dCH (phenyl)	0,09249	0,47912
46:	1023,42	dCC (phenyl)	0,47547	117,84306
47:	1026,54	dCH (phenyl)	0,14625	0,20281
48:	1051,01	dCH (phenyl)	8,22837	30,98032
49:	1059,65	vCN (N14+C26)	1,43306	25,98084
50:	1070,11	complex	35,81992	10,44693
51:	1081,92	complex	11,61723	1,57312
52:	1104,94	vCC (C11+C12)	18,10655	12,52934
53:	1112,27	complex	34,15816	7,89083
54:	1138,66	complex	50,78065	0,88049
55:	1151,95	vCN (N4+C11)	21,93683	6,61227
56:	1174,62	dCH (phenyl)	0,1098	7,50825
57:	1198,16	dCH (phenyl)	0,9063	7,8858
58:	1229,04	dNH	26,2925	27,78926
59:	1231,02	vCC (C27+C28)	3,09646	74,21159
60:	1262,42	complex	12,18084	18,97658
61:	1288,61	complex	1,46669	34,639
62:	1315,89	complex	15,90837	6,03345
63:	1337,80	vCC (phenyl)	0,28834	7,26834
64:	1354,84	dCH (methyne C11)	140,60012	21,42372
65:	1363,40	dCH (phenyl)	12,51044	10,2687
66:	1375,81	dCH (methyl C12)	114,48041	5,12978
67:	1384,18	complex	1,8319	37,50757
68:	1407,00	vCOO	249,01984	12,49141
69:	1413,88	dCH (methyne C26)	29,47335	7,81409
70:	1477,61	vCH (methylene C27)	21,55962	14,0894
71:	1479,14	dCH (methyl C12)	50,95165	9,62248
72:	1484,24	dCH (methyl C12)	23,26804	8,13615
73:	1486,02	dNH_3	170,71765	12,26812
74:	1489,67	dNH_3	151,52877	4,03058
75:	1530,10	dCH (phenyl)	21,4813	1,57963
76:	1537,73	dNH	705,68238	12,25732
77:	1609,62	dNH_3	216,66707	12,32525
78:	1614,02	vCOO	725,5488	2,11027
79:	1622,81	vCC (phenyl)	4,68277	31,36604
80:	1637,82	dNH_3	42,90014	11,62835
81:	1642,50	vCC (phenyl)	4,51782	103,44673
82:	1686,36	vCO	512,95874	17,11683
83:	3036,74	vCH (methyl C12)	29,07311	417,99449

84:	3058,53	vCH (methylene C27)	14,09711	321,91107
85:	3064,47	vCH (methyne C11)	20,47461	206,69868
86:	3105,33	vCH (methyl C12)	38,87831	152,76617
87:	3106,09	vCH (methylene C27)	4,92368	161,07217
88:	3121,43	vCH (methyl C12)	30,42908	182,91396
89:	3129,07	vCH (methyne C26)	6,55652	112,67437
90:	3163,61	vCH (phenyl)	7,50296	134,33697
91:	3165,94	vCH (phenyl)	6,91628	169,93553
92:	3177,21	vCH (phenyl)	6,02111	297,06467
93:	3186,34	vCH (phenyl)	30,47753	93,68657
94:	3195,96	vCH (phenyl)	15,15285	784,36718
95:	3369,83	vNH_3	64,53454	234,9966
96:	3444,43	vNH_3	313,45643	137,92389
97:	3503,92	vNH_3	192,09046	76,49323
98:	3518,59	vNH	226,92915	162,68254

Table S3. *Proposed assignment and theoretical frequencies of IR and Raman normal modes calculated using the B3LYP functional for the l-zwi(PheAla) isolated molecule embedded in a dielectric environment.*

The region between 1250 and 1650 cm^{-1} is characterized by very significant differences between measurements collected at room temperature on the pristine sample (*l*-PheAla) and on the residual powder after heating at the working temperature for mass spectrometry measurements (*r*-PheAla). Such differences are very well reproduced by theoretical calculations of *l*-zwi and *c*-neu structures, respectively, as shown in Figure S2.

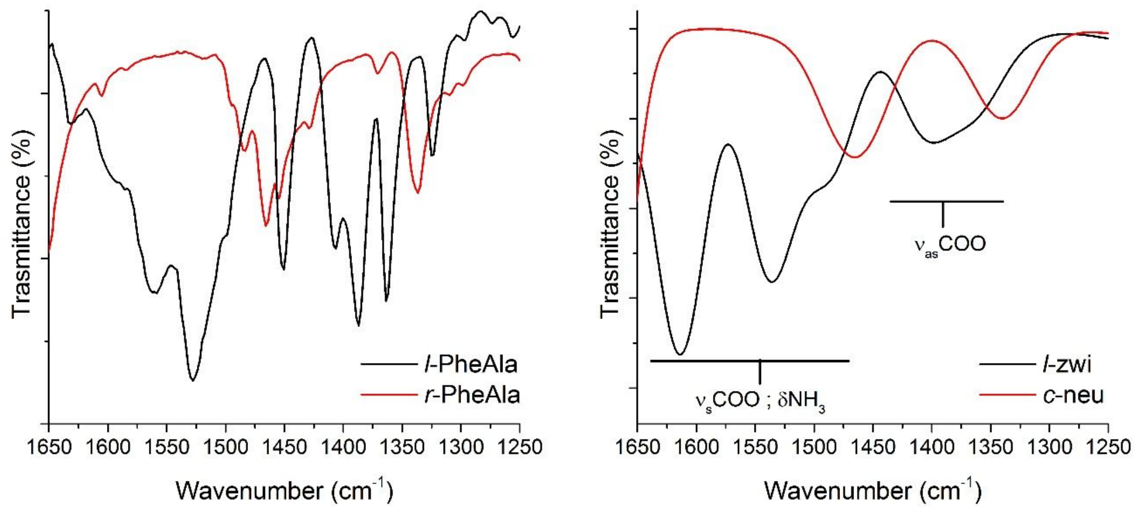

Figure S2. *Comparison between the experimental IR spectra of l-PheAla (black curve) and r-PheAla (red curve) and between the simulated IR spectra of l-zwi (black) and c-neu (red) structures, on the left and right side respectively, in the range 1650-1250 cm^{-1}. The main contribution of COO and NH_3 vibrations is indicated.*

Before heating, several contributions are grouped in large spectral features. The simulations of the *l*-zwi structure (black curve) suggests that the high energy band, roughly between 1450 and 1650 cm^{-1}, receives contributions from the asymmetric stretching of the terminal COO group (1614 cm^{-1}) and three groups of bands involving NH_3 bending (1610, 1490 and 1486 cm^{-1}), NH bending

(1538 cm^{-1}) and Phenyl CH bending (1490 and 1486 cm^{-1}), as also detailed in Table S3. A weaker and lower-energy group of bands between 1300 and 1450 cm^{-1} contains as main contributions the symmetric stretching of the COO group (1407 cm^{-1}), accompanied by CH bending modes of the methyl (1376 cm^{-1}) and methyne (1355 cm^{-1}) groups. These contributions are drastically reduced after cyclization, due to the very different dynamical behavior of the six-membered ring, where the COO group is not present anymore, with respect to the linear structure. In the *c*-neu simulation (red curve) the former among two surviving bands can be assigned to the peculiar stretching mode of CC and CN bonds in the diketopiperazine ring (1452 cm^{-1}), accompanied by the CH bending of methylene (1476 cm^{-1}) and methyl (1478 cm^{-1}, reinforced by the interaction with the -CH_2-Phenyl group, that in the case of *c*-neu tends to fold toward CH_3) groups. The latter band is also dominated by CC and CN stretching modes (1347 cm^{-1}) involving both diketopiperazine and phenyl rings.

The experimental IR measurements collected at room temperature on the *c*-GlyPhe and *c*-AlaGly samples are reported and compared with the results obtained on the *r*-PheAla sample. In the calculations the cyclo neutral (*c*-neu) structures of the isolated PheAla molecule and the *c*-neu structures of GlyPhe and AlaGly molecules have been considered. The comparison between experimental and theoretical results is shown in Figure S3.

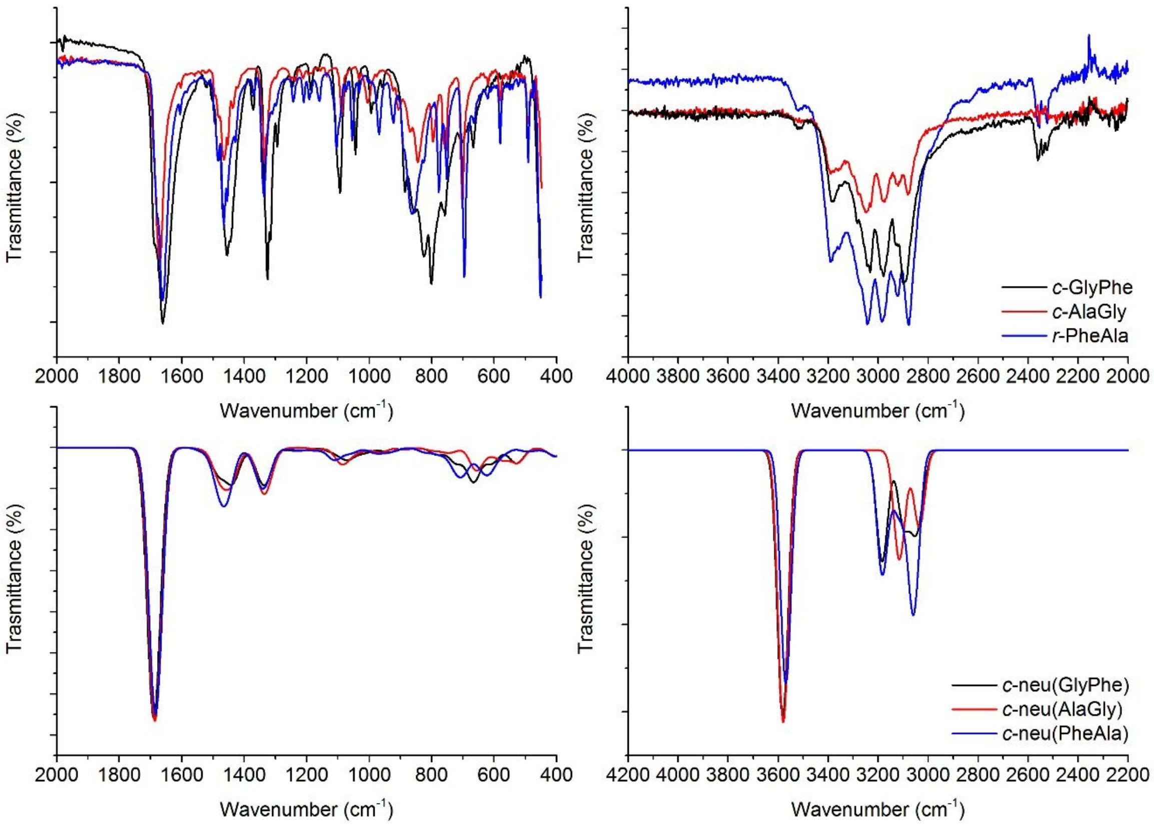

Figure S3. *Top: comparison between the IR spectra measured at room temperature on c-GlyPhe (black curve), c-AlaGly (red curve) and r-PheAla (blue curve) in the range 2000-400 cm^{-1} and 4000-2000 cm^{-1} on the left and right panel, respectively. Bottom: comparison between the simulated IR spectra calculated for the cyclo neutral structure of GlyPhe (black curve), AlaGly (red curve) and PheAla (blue curve) in the range 2000-400 cm^{-1} and 4200-2200 cm^{-1} on the left and right panel, respectively. The blue shift observed*

in the frequency range above 2000 cm⁻¹ between the experimental and simulated spectra is due to the lack of anharmonic contributions and intermolecular H-bonds in the simulations[5-8].

The full vibrational modes of *c*-neu(GlyPhe) and *c*-neu(AlaGly) are listed in Table S4.

***c*-neu(GlyPhe) normal modes analysis**			***c*-neu(AlaGly) normal modes analysis**		
**Mode**	**Wavenumber (cm^{-1})**	**IR activity (a.u.)**	**Mode**	**Wavenumber (cm^{-1})**	**IR activity (a.u.)**
6:	45,04	0,2258	6:	52,45	13,54239
7:	61,73	0,097	7:	105,24	6,33952
8:	74,96	0,1985	8:	161,62	2,13788
9:	96,00	17,00602	9:	233,64	0,12923
10:	116,05	2,55213	10:	254,46	0,93908
11:	184,55	5,20369	11:	394,95	23,19883
12:	195,22	0,27894	12:	411,32	37,04999
13:	264,18	2,72556	13:	454,34	0,77555
14:	368,13	0,54978	14:	472,14	1,87051
15:	405,13	53,0136	15:	524,36	95,10715
16:	418,24	3,23582	16:	571,93	71,06056
17:	423,46	1,31924	17:	609,66	33,98473
18:	454,67	2,91199	18:	657,34	146,00219
19:	476,79	2,95128	19:	733,56	22,52317
20:	504,55	5,79904	20:	759,32	18,17539
21:	528,51	95,39483	21:	799,39	17,41171
22:	563,78	12,10273	22:	888,70	14,14577
23:	606,55	88,71089	23:	963,61	31,43184
24:	623,87	15,23978	24:	1014,02	14,73897
25:	637,90	0,30617	25:	1050,77	29,94079
26:	666,28	218,57755	26:	1086,17	94,30288
27:	720,04	54,15309	27:	1104,86	11,69155
28:	723,51	43,77334	28:	1166,70	10,47402
29:	770,79	18,10676	29:	1265,29	1,69709
30:	782,94	18,57272	30:	1319,62	103,81002
31:	794,68	13,99189	31:	1331,80	4,08854
32:	854,54	3,1818	32:	1341,72	228,54452
33:	867,93	0,28274	33:	1393,09	2,14273
34:	924,70	17,07631	34:	1397,82	11,31008
35:	937,62	8,84415	35:	1440,93	197,75626
36:	956,57	9,22128	36:	1468,89	4,90135
37:	990,08	8,65776	37:	1472,86	128,68715
38:	1005,05	0,96612	38:	1479,45	6,75812
39:	1019,23	22,93121	39:	1488,80	67,7029
40:	1023,85	6,67319	40:	1534,70	8,33793
41:	1025,09	0,09625	41:	1685,17	1357,96948
42:	1051,74	5,54694	42:	1689,58	426,03558
43:	1067,78	64,94414	43:	3033,18	29,00059
44:	1100,00	30,13864	44:	3038,74	18,31463
45:	1131,63	13,36634	45:	3088,37	6,34262
46:	1172,82	0,09997	46:	3112,54	33,9636

47:	1197,55	1,39341	47:	3117,92	5,63755
48:	1200,51	8,84732	48:	3121,42	25,71863
49:	1229,91	13,25605	49:	3576,47	91,81647
50:	1255,52	6,26228	50:	3583,86	77,21065
51:	1262,78	3,85015			
52:	1320,89	64,23782			
53:	1333,49	72,74081			
54:	1343,75	125,00946			
55:	1366,36	10,59319			
56:	1370,06	5,26878			
57:	1390,24	9,88883			
58:	1436,71	214,80389			
59:	1465,55	2,70103			
60:	1474,52	65,36791			
61:	1485,28	61,56009			
62:	1487,47	43,96847			
63:	1530,20	15,7656			
64:	1532,29	8,07372			
65:	1621,43	0,77656			
66:	1643,17	7,10418			
67:	1689,38	1680,19287			
68:	1692,19	78,93949			
69:	3031,60	24,98794			
70:	3056,84	33,25957			
71:	3093,53	23,50367			
72:	3103,93	16,34658			
73:	3113,77	6,05199			
74:	3165,99	4,46456			
75:	3169,07	0,75479			
76:	3177,26	13,66529			
77:	3184,46	38,08509			
78:	3194,48	14,88901			
79:	3576,55	85,15406			
80:	3587,67	83,91374			

Table S4. *Theoretical frequencies of IR normal modes calculated using the B3LYP functional for the c-neu(GlyPhe) and c-neu(AlaGly) isolated molecules, on left and right, respectively. IR activity stands for the squared modulus of the transition dipole moment.*

The comparison between the simulated and experimental IR spectra showed in Figure S3 indicates that these cyclo dipeptides are characterized by the same fingerprints of the simulated spectrum for *c*-neu(PheAla), and the measured spectrum of *r*-PheAla. The analysis of vibrational IR bands is consistent with the predictions for *c*-neutral structures of these molecules. Measurement and simulation of IR spectra of *c*-AlaGly and *c*-GlyPhe, show striking similarities with *r*-PheAla. In the following, the characteristic IR vibrational modes compatible with a cyclo-dipeptide structure are reported and compared for the three samples.

	**IR experimental frequencies (cm^{-1})**

Assignment	*c*-AlaGly	*c*-GlyPhe	*r*-PheAla
NH	3320	3310	3315
CH (phenyl)	3025-3100	3025-3100	3150-3200
CH (alkyl)	2800-3025	2800-3025	2900-3150
CO	1660 (C=O)	1660 (C=O)	1660 (C=O)
	**Raman experimental frequencies (cm^{-1})**		
**Assignment**	***c*-AlaGly**	***c*-GlyPhe**	***r*-PheAla**
CO	1656 (C=O)	1661 (C=O)	≈1650 (C=O)
CC & CN (DKP)	1523	1521	1520
NH	*absent*	*absent*	*absent*
Complex skeletal mode (phenyl & DKP)	764	764	763
CN (DKP)	≈470	≈480	458 ; 474
CC (phenyl & DKP)	*absent*	≈500	493

Table S5. *Proposed assignment and experimental frequencies of some of the main IR (top) and Raman (bottom) bands diagnostic for the identification of cyclo structures of GlyAla, GlyPhe and PheAla molecules*[9-14].

The assignment in the PheAla Raman spectra of the characteristic lines of the phenyl-CH_2- group of the structures *l*-neu, *l*-zwi and *c*-neu (see figure S4) has been done by a close comparison between the spectra of *c*-GlyPhe (containing the phenyl-CH_2- group) and *c*-AlaGly (not containing the group).

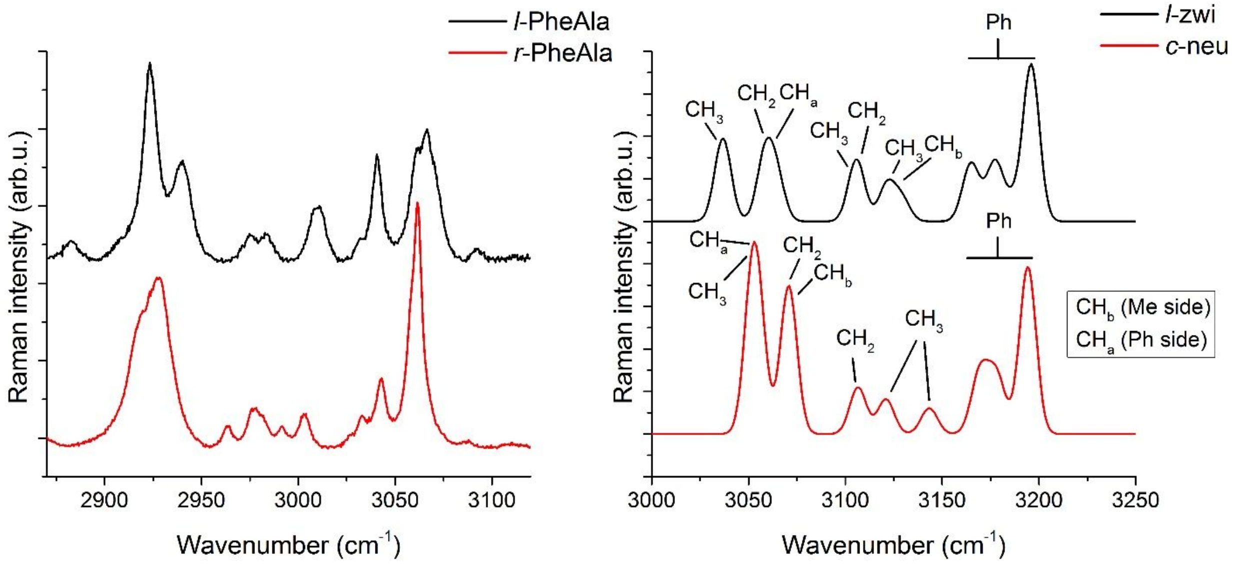

Figure S4. *Left panel: Raman comparison between the experimental spectra of l-PheAla (black curve) and r-PheAla (red curve) samples in the range 2870-3120 cm^{-1}. Right panel: Raman comparison between the simulated spectra of l-zwi (black curve) and c-neu (red curve) structures of PheAla specie in the range 3000-3250 cm^{-1}. The characteristic lines of the phenyl-CH_2- group are reported. We choose to show the simulated spectra in the region 3000-3250 cm^{-1} of the CH vibrations shifted by 130 cm^{-1} to better compare with the experimental results. This blue shift in the high frequency region of the simulated spectra is due to the lack of anharmonic contributions and intermolecular H-bonds (see main text, section 3).*

The comparison between the experimental Raman spectra of *r*-PheAla, *c*-AlaGly and *c*-GlyPhe samples and the simulated spectra of *c*-neu(PheAla), *c*-neu(AlaGly) and *c*-neu(GlyPhe) is shown in Figure S5.

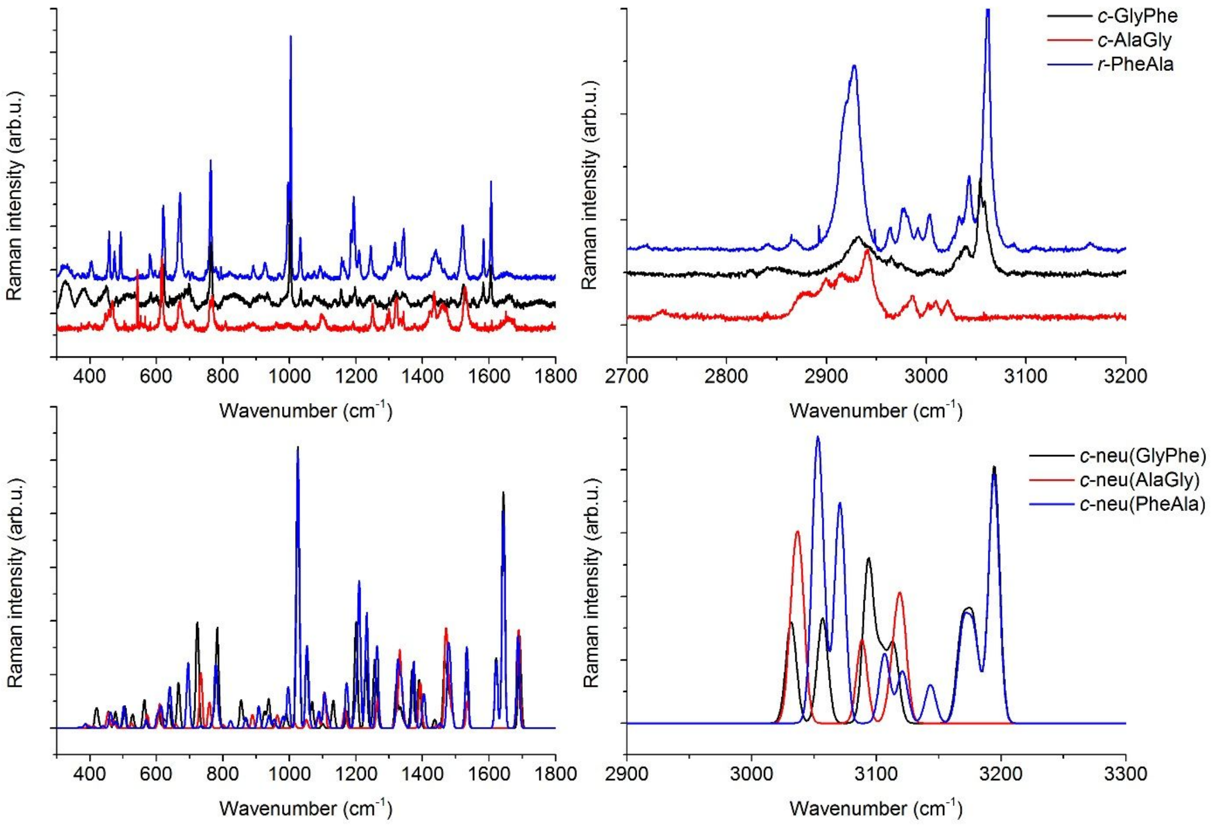

Figure S5. *Top: Comparison of the room temperature Raman spectra of the r-PheAla sample (blue curve) and the other cyclo species, c-GlyPhe (black curve) and c-AlaGly (red curve) in the range 300-1800 cm^{-1} and 2700-3200 cm^{-1} on the left and right panel, respectively. Bottom: comparison between the simulated Raman spectra calculated for the neutral cyclic structure of GlyPhe (black curve), AlaGly (red curve) and PheAla (blue curve) in the range 300-1800 cm^{-1} and 2900-3300 cm^{-1} on the left and right panel, respectively. The blue shift observed in the frequency range above 2000 cm^{-1} between the experimental and simulated spectra is due to the lack of anharmonic contributions and intermolecular H-bonds in the simulations.*

3. Theoretical results on the GlyGly and PheAla cyclization mechanisms

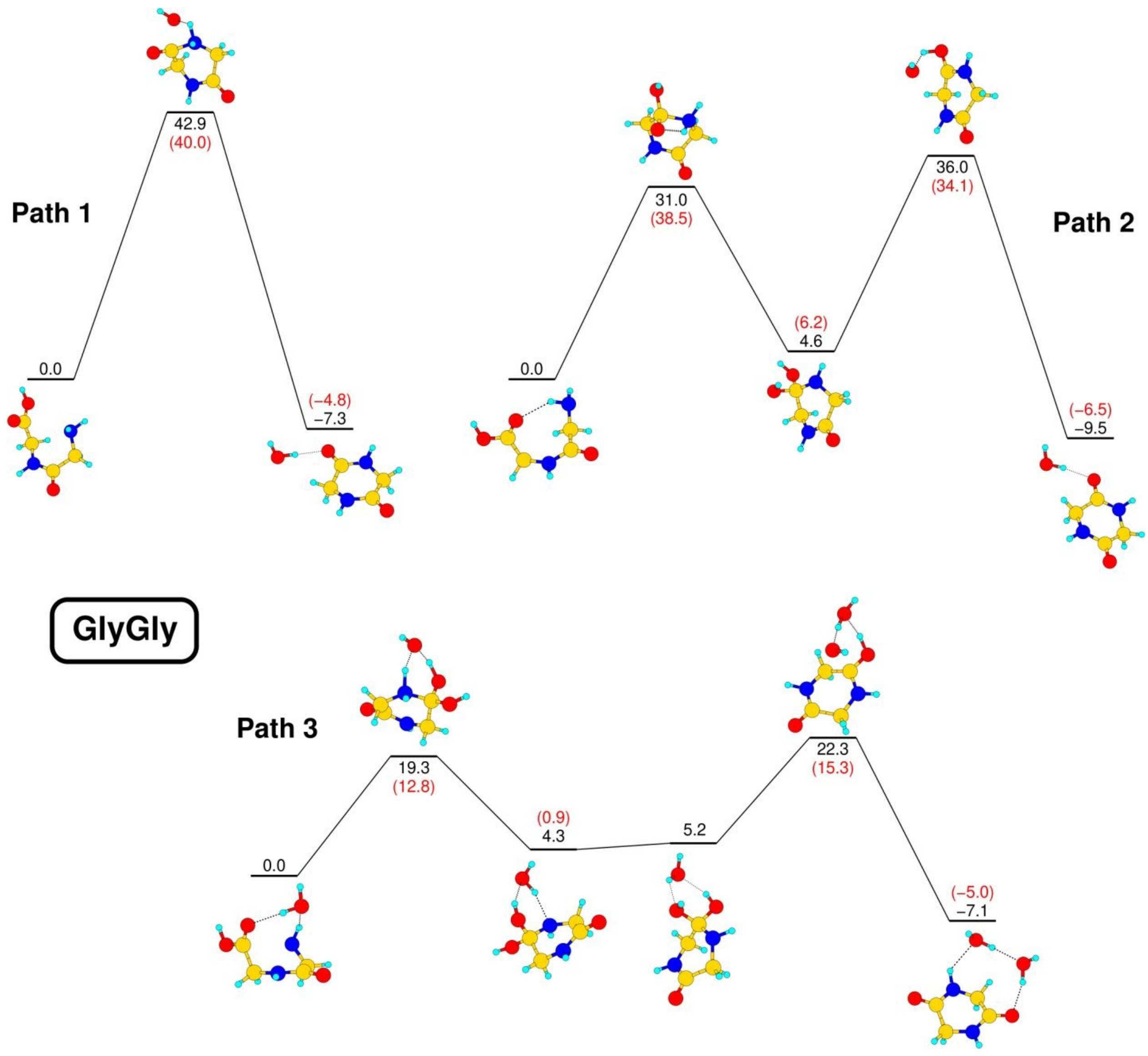

Figure S6. *Optimized structures of stationary points of intramolecular (Paths 1 and 2) and intermolecular (Path 3) cyclization reactions of GlyGly. Total energy values, calculated in a M06-2X@def2-TZVPP framework, are expressed in kcal/mol.*

Figure S6 summarizes the results of a preliminary investigation of the reaction barriers applied to the cyclization of a linear, neutral GlyGly molecule, already investigated in a previous study[15]. We note that stable structures and reaction paths have been obtained independently from such reported by Li et al., which are also indicated in Figure S6, printed in red. Despite of the indipendent approach and the slighty different theoretical framework used in the two studies, the results are qualitatively very similar, and show a quantitative agreement close enough to provide a solid ground to apply the same method to the cyclization of PheAla, not investigated in previous studies.

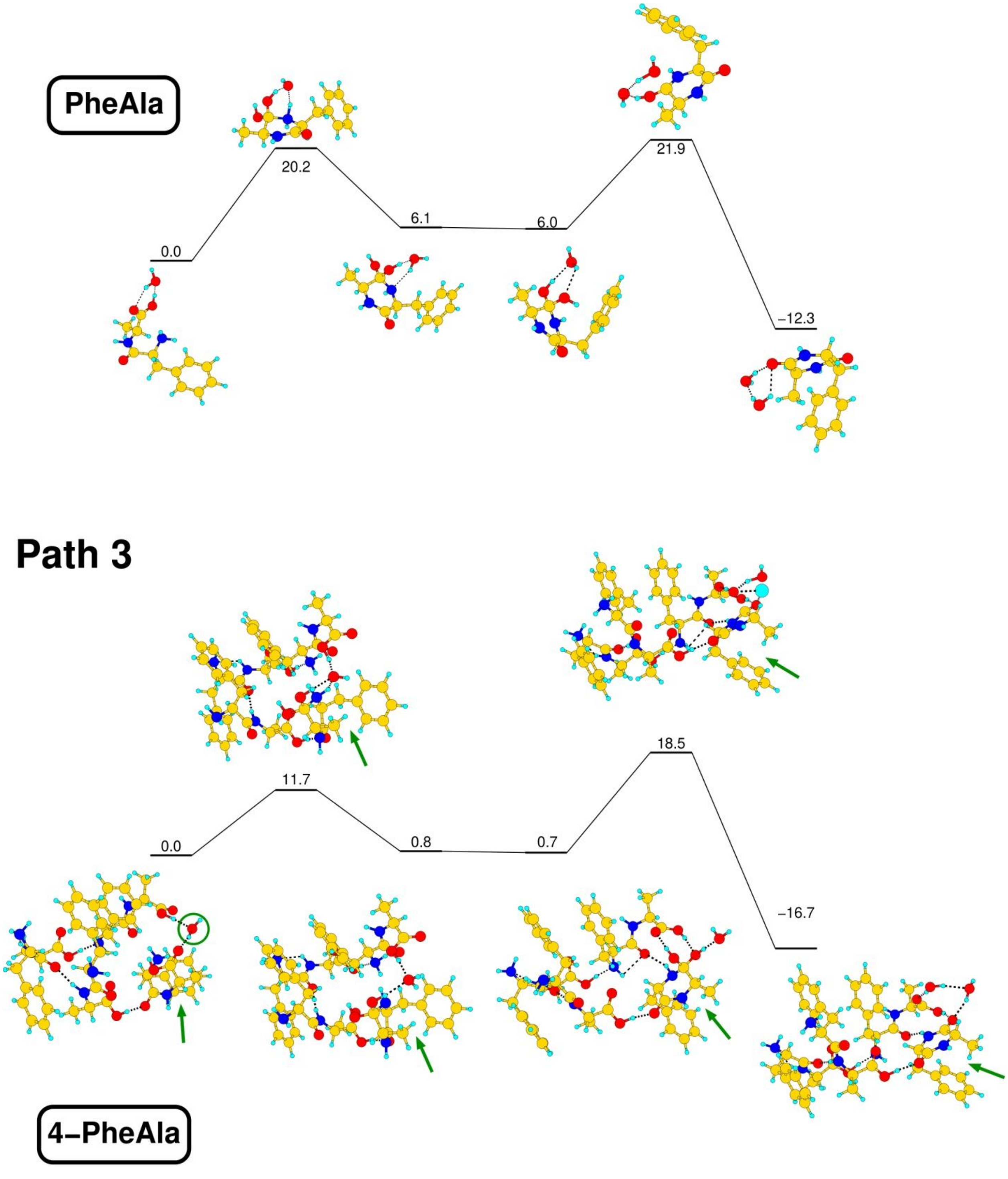

Figure S7. *Reaction coordinates and barriers for water-catalyzed l-PheAla cyclization in the case of one (upper part) and four interacting (lower part) linear dipeptides. The calculations have been performed at the r²-SCAN-3c@mTZVPP level of theory, as discussed in the text. In the lower part of the figure, for the sake of clarity the l-PheAla molecule undergoing cyclization is indicated by a green arrow, while the catalytic water molecule is enclosed in a green circle in the first step.*

A preliminary analysis of the effect of the molecular aggregation of *l*-PheAla in the solid state, and of its effect on dipeptide cyclization, has been performed and will be briefly discussed here. We focus on a system containing four *l*-PheAla molecules and only one water molecule (see Figure S7), thus investigating only effects due to inter-peptide interactions and limiting the fluctuation due to an enhanced catalytic effect of the latter species. A first assessment of the system has been obtained using the xTB-GFN2 Hamiltonian and the CREST sorting tool, as discussed in the main text. However, DFT investigation at the M062X@def2-TZVPP level of theory is quite expensive in terms of computational resources. For this reason, we fell back to the robust r^2-SCAN-3c functional with its tailored mTZVPP basis set[16], whose application to the one-PheAla+H_2O mechanism (see Figure S7, upper part) provides results close to those obtained using M062X and reported in Figure 7 of the main text. Regarding the four-PheAla+H_2O mechanism (Figure S7, lower part), the water catalyst finds a third anchoring point in a strong H bond with a neighboring -COOH group, leading to a significant lowering of the barrier to cyclization, accompanied to the formation of the -$C(OH)_2$ intermediate. No particular effect is reported in the case of the reaction barrier leading to the water-catalyzed elimination of a second water molecule from the -$C(OH)_2$ intermediate. This is likely due to the fact that this second reaction occurs on the outskirts of the simple four-molecule system employed, where the catalyst is not anymore coordinated to a neighboring dipeptide molecule, leading to results in line with previous findings. These results also suggest that a more complex and thick network of interactions, whose theoretical representation is clearly beyond the scope of this preliminary study, is required to theoretically unravel the massive rearrangement process which takes place when the real sample is heated at 85 °C.

References

1 Ziganshin, M. A.; Larionov, R. A.; Gerasimov, A. V.; Ziganshina, S. A.; Klimovitskii, A. E.; Khayarov, K. R.; Mukhametzyanov, T. A.; Gorbatchuk, V. V. Thermally induced cyclization of L-isoleucyl-L-alanine in solid state: Effect of dipeptide structure on reaction temperature and self-assembly. *J. Pep. Sci.*, **2019**, *25*, e3177.

2 Ziganshin, M.A.; Safiullina, A.S.; Gerasimov, A.V.; Ziganshina, S.A.; Klimovitskii, A.E.; Khayarov, K.R.; Gorbatchuk, V.V. Thermally induced self-assembly and cyclization of L-Leucyl-L-Leucine in solid state. *J. Phys. Chem. B,* **2017**, *121*, 8603-8610.

3 Ziganshin, M.A.; Gerasimov, A.V.; Ziganshina, S.A.; Gubina, N.S.; Abdullina, G.R.; Klimovitskii, A.E.; Gorbatchuk, V.V.; Bukharaev, A.A. Thermally induced diphenylalanine cyclization in solid phase. *J. Therm. Anal. Calorim.*, **2016**, *125*, 905-912.

4 Amdursky, N.; Beker, P.; Koren, I.; Bank-Srour, B.; Mishina, E.; Semin, S.; Rasing, T.; Rosenberg, Y.; Barkay, Z.; Gazit, E. et al. Structural transition in peptide nanotubes. *Biomacromolecules*, **2011**, *12*, 1349-1354.

5 Chaban, G.M.; Gerber, R.B. Anharmonic vibrational spectroscopy calculations with electronic structure potentials: comparison of MP2 and DFT for organic molecules. *Theor. Chem. Account*, **2008**, *120*, 273-279

6 Bloino, J.; Biczysko, M.; Barone, V. Anharmonic effects on vibrational spectra intensities: infrared, Raman, vibrational circular dichroism, and Raman optical activity. *J. Phys. Chem. A*, **2015**, *119*, 11862-11874.

7 Howard, A.A.; Tschumper, G.S.; Hammer, N.I. Effects of hydrogen bonding on vibrational normal modes of pyrimidine. *J. Phys. Chem. A*, **2010**, *114*, 6803-6810.

8 Barth, A. Infrared spectroscopy of proteins. *Biochim. Biophys. Acta*, **2007**, *1767*, 1073-1101.

9 Tul'chinskii, V.M.; Miroshnikov, A.I.; Kostetskii, P.V.; Kogan, G.A. Spectra in the middle and far IR regions of cyclic peptide compounds with a cis amide group. *Chem. Nat. Compd.*, **1973**, *9*, 745-751.

10 Koleva, B.B.; Kolev, Ts.; Zareva, S.Y.; Spiteller, M. The dipeptide alanylphenylalanine (H-Ala-Phe-OH) – protonation and coordination ability with Au(III). *J. Mol. Struct.,* **2007**, *831,* 165-173.

11 Mahalakshmi, R.; Jesuraja, S.X.; Das, S.J. Growth and characterization of L-phenylalanine. *Cryst. Res. Technol.*, **2006**, *41*, 780-783.

12 Olsztynska, S.; Komorowska, M.; Vrielynck, L.; Dupuy, N. Vibrational spectroscopic study of L-Phenylalanine: effect of pH. *Appl. Spectrosc.*, **2001**, *55*, 901-907.

13 Gangopadhyay, D.; Sharma, P.; Singh, S.K.; Singh, P.; Tarcea, N.; Deckert V.; Popp, J.; Singh, R.K. Raman spectroscopic approach to monitor the in vitro cyclization ofcreatine→ creatinine. *Chem. Phys. Lett.,* **2015**, *618,* 225–230.

14 Hernández, B.; Pflüger, F.; Kruglik, S.G.; Ghomi, M. Characteristic Raman lines of phenylalanine analyzed by a multiconformational approach. *J. Raman Spectrosc.*, **2013**, *44*, 827-833.

15 Li, Y.; Li, F.; Zhu, Y.; Li, X.; Zhou, Z.; Liu, C.; Zhang, W.; Tang, M. DFT study on reaction mechanisms of cyclic dipeptide generation. *Struct. Chem.*, **2016**, *27*, 1165-1173.

16 Grimme, S.; Hansen, A.; Ehlert, S.; Mewes, J.M. r2SCAN-3c: A "Swiss army knife" composite electronic-structure method, *J. Chem. Phys.*, **2021**, *154*, 064103.
